# Meta-analysis of GWAS in canola blackleg (*Leptosphaeria maculans*) disease traits demonstrates increased power from imputed whole-genome sequence

**DOI:** 10.1038/s41598-020-71274-6

**Published:** 2020-08-31

**Authors:** M. Fikere, D. M. Barbulescu, M. M. Malmberg, G. C. Spangenberg, N. O. I. Cogan, H. D. Daetwyler

**Affiliations:** 1 School of Applied Systems Biology, La Trobe University, Bundoora, VIC 3086 Australia; 2 Centre for AgriBioscience, Agriculture Victoria, AgriBio, Bundoora, VIC 3083 Australia; 3 Queensland Alliance for Agriculture and Food Innovation (QAAFI), The University of Queensland, Brisbane, QLD 4072 Australia; 4 Agriculture Victoria, Grains Innovation Park, Horsham, VIC 3401 Australia

**Keywords:** Data processing, Gene ontology, Genome informatics, Quality control, Statistical methods, Agricultural genetics, Genetic association study, Genetic markers, Heritable quantitative trait, Plant breeding, Plant genetics, Quantitative trait, Sequencing, Genetics

## Abstract

Blackleg disease causes yield losses in canola (*Brassica napus* L.). To identify resistance genes and genomic regions, genome-wide association studies (GWAS) of 585 diverse winter and spring canola accessions were performed using imputed whole-genome sequence (WGS) and transcriptome genotype-by-sequencing (GBSt). Blackleg disease phenotypes were collected across three years in six trials. GWAS were performed in several ways and their respective power was judged by the number of significant single nucleotide polymorphisms (SNP), the false discovery rate (FDR), and the percentage of SNP that validated in additional field trials in two subsequent years. WGS GWAS with 1,234,708 million SNP detected a larger number of significant SNP, achieved a lower FDR and a higher validation rate than GBSt with 64,072 SNP. A meta-analysis combining survival and average internal infection resulted in lower FDR but also lower validation rates. The meta-analysis GWAS identified 79 genomic regions (674 SNP) conferring potential resistance to *L. maculans*. While several GWAS signals localised in regions of known Rlm genes, fifty-three new potential resistance regions were detected. Seventeen regions had underlying genes with putative functions related to disease defence or stress response in *Arabidopsis thaliana*. This study provides insight into the genetic architecture and potential molecular mechanisms underlying canola *L. maculans* resistance.

## Introduction

Canola (also known as rapeseed, *Brassica napus* L., genome AACC, 2n = 38) is one of the most important sources of edible oil providing 14% of global production^1^. Strong canola growth demand requires critical increases in overall production and productivity, which can in part be achieved through overcoming biotic and abiotic stress. One of the major biotic stressors is blackleg disease, which makes it a high priority trait for canola breeding programs. It is caused by the fungal pathogen *Lepthosphaeria maculans* resulting in large yield losses, thereby becoming a major threat to world canola production. Most canola growing regions experience losses due to blackleg disease, with Canada and France having reported between 5 and 40% average yield losses annually^2,3^. Similar yield reduction is common in Australia, however, in some regions losses as severe as 90% have been observed^4–6^. Genetic resistance to blackleg can be either qualitative (race specific) or quantitative (non-race specific) and commercial cultivars tend to have qualitative resistance genes that, when effective, confer complete immunity against blackleg. However, quantitative resistance has the potential to only restrict the development of *L. maculans* formation throughout plant development stages^7–10^.

Elucidating the genetic basis of blackleg resistance as well as identification and localization of resistance genes/genomic regions to *L. maculans* is important to understand the genetic architecture of resistance to the pathogen and is an important step to mitigate its catastrophic effect. Early studies focused on bi-parental mapping of quantitative trait loci (QTL) using RFLP, SSR, AFLP, RAPD or DArT markers for blackleg disease resistance^11–23^. In addition, several environmentally stable QTL were identified and localized in A and C sub-genomes conferring resistance to *L. maculans*^24–26^. Moreover, assembling pangenomes has been applied to discover novel genes in various species^27,28^. A study by Hurgobin et al.^29^ pointed out that one of the causes for gene absence or presence in *B. napus* was homoeologous exchange and it contributed towards resistance gene diversity.

In the past decade, SNPs have become the genetic marker of choice over multi-allelic markers due to their abundance in the genome and high polymorphism rate^30^. Raman et al.^31^ used SNP-chip based linkage maps and found a major *L. maculans* resistance locus on chromosome A07 that accounted for up to 69.2% of genetic variation and was mapped around the major locus Rlm4. While the genetic variance explained with bi-parental populations is often estimated to be high, these loci explain much less of the variation generally observed in more diverse germplasm. Fikere et al.^32^ demonstrated that a large proportion of genetic variation for blackleg disease resistance in diverse canola population remains unexplained by currently published QTL studies. Nevertheless, bi-parental QTL mapping experiments were an important first step towards increasing the understanding of blackleg disease architecture in canola.

The development of advanced sequencing and SNP chip technologies, and the associated drop in genotyping prices, has enabled the use of thousands of SNP markers across the genome. The increased SNP density allows for the application of single SNP regressions or genome-wide association studies (GWAS) in more diverse germplasm. Genome-wide association studies have rapidly increased the discovery of new putative candidate genes for complex traits in a variety of crops such as rice^33^, maize^34^, soybean^35^, and flax^36,37^. In canola, several GWAS have been reported for agronomic and quality traits. Few GWAS have been reported on major canola diseases (*Leptosphaeria maculans* and *Sclerotinia* stem rot)^22,38,39^. Most recently, Raman et al.^31^ used a panel of 179 diverse canola lines genotyped with 18,804 SNPs to perform a GWAS that discovered a new *L. maculans* resistance gene Rlm12 on chromosome A01 as well as additional QTL.

We investigated two main ways to increase the power of GWAS using (1) meta-analysis of single trait GWAS to identify pleiotropic loci and (2) imputed whole-genome sequence (WGS) data. A meta-analysis GWAS combines single trait results from different traits or different environments^40,41^. This would increase the power to uncover pleiotropic loci affecting multiple traits. The increased power has been demonstrated in several studies in human and the meta-analyses have been applied widely in human and animal genetics as they avoid sharing of original genotypes and phenotypes^40–44^.

The use of WGS variants substantially increases the number of markers and thus increases linkage disequilibrium (LD) between markers and causative mutations. Furthermore, the causative mutations may themselves be genotyped directly. This is proposed to lead to more effective pinpointing of genes or genomic regions linked with complex traits of interest. However, WGS for a large number of individuals is expensive. A more cost-effective approach is to impute WGS into individuals that have been genotyped with a lower density assay. A number of imputation software programs are available, such as MACH^45^, IMPUTE^46^, BEAGLE^47^, FImpute^48^, and Eagle^49^ + Minimac^50^, and they vary in computational efficiency and imputation accuracies^51,52^. Imputation has been implemented in several crops, a study by Torkamaneh et al.^53^ achieved high accuracy when imputing from low-coverage genotyping-by-sequencing (GBS) to WGS in soybean. Similarly, Shi et al.^54^ examined the potential of exome sequence imputation in wheat. These studies emphasized the need to carefully evaluate imputation algorithms in new datasets as large differences in performance exist.

Here we report on an imputation analysis from transcriptome GBSt to WGS and GWAS in a relatively large population of spring and winter canola phenotyped across three growing seasons. We impute WGS into 585 canola lines genotyped with GBSt and analyse this dataset with single-trait GWAS with the aim of: (i) comparing the power of GWAS using GBSt and imputed WGS and (ii) performing meta-analysis of GWAS for two blackleg resistance traits (survival and internal infection) and identify genomic regions potentially harbouring new resistance genes associated with blackleg traits as well as compare its power to single-trait GWAS.

## Results

### Phenotypic variation and correlation coefficient

In-field phenotypes for 585 diverse canola lines in field trials from 2015 to 2017 were adjusted for spatial variation and best linear unbiased estimates (BLUEs) were calculated per trial and per line (Table 1). Performance of emergence and internal infection varied across the 2015–2017 growing seasons. For instance, in the 2015 trial, seedling emergence counts were substantially lower at Green Lake than Wickliffe, possibly due to drier conditions at Green Lake. Blackleg traits (survival rate and average internal infection) also varied across sites. As expected, in the blackleg managed 2016 and 2017 trials average internal infection percentages were lower (range 7.22–19.2) than in the 2015 blackleg disease nurseries. Broad sense heritability of the three traits ranged from 0.38 (emergence count) at Mininera to 0.80 (survival rate) at the Wickliffe site (Table 1).

**Table 1:** Phenotypic summary after spatial adjustment of the three traits in the association panel across environments during 2015–2017 growing seasons for 585 winter and spring lines in 2015, and 168 spring lines grown in 2016 and 2017.

Year	Locations	Trait	Mean	SD	${\text{H}}^{2}$
2015	WL15	Emergence count	31.24	11.13	0.46
		Survival rate	22.5	16.96	0.80
		AvInf	84.1	12.54	0.77
	GL15	Emergence count	14.44	3.49	0.42
		Survival rate	56.03	15.05	0.54
		AvInf	57.04	16.9	0.74
2016	MI16	Emergence score	5.24	0.31	0.38
		AvInf	19.2	4.39	0.76
	HrI16	Emergence score	6.41	0.56	0.44
		AvInf	7.22	4.44	0.68
2017	Hr17	Emergence score	5.37	1.12	0.45
		AvInf	11.36	3.18	0.67
	HrI17	Emergence score	4.35	0.3	0.42
		AvInf	17.48	5.56	0.72

AvInf = Average internal infection, WL15 = Wickliffe, GL15 = Green Lake, MI16 = Mininera, HrI16 = Horsham irrigated 2016, HrI17 = Horsham irrigated 2017, Hr17 = Horsham rain-fed 2017 and ${\text{H}}^{2}$ = broad sense heritability.

The phenotypic correlations of BLUEs between three traits at 5 locations (Wickliffe, Green Lake, Mininera, Horsham irrigated 2016 and 2017, and Horsham rain-fed) in three years (2015, 2016, 2017) are shown in Supplementary Fig. S1. Moderately positive correlation coefficients were observed between similar traits at different sites and years. A strong negative correlation coefficient was recorded between average internal infection and survival rate (r =  − 0.86), following the expected trend of high infection being associated with low survival (Supplementary Fig. S1).

### Genomic data and imputation accuracy

We evaluated the imputation accuracy (correlation of observed and imputed genotypes) and concordance rate of WGS in 153 spring and winter type canola lines (Supplementary Table S1a). The low-density transcriptome genotyping-by-sequencing (GBSt) of these 153 lines consisted of 64,072 SNPs that overlapped with the 6 million WGS variants. Testing included FImpute and Eagle + Minimac. The correlation between observed and imputed genotypes was considerably higher in Eagle + Minimac3 compared to FImpute in all the sub-population validation scenarios (Supplementary Table S1a). For instance, the correlations for Eagle + Minimac3 imputed spring lines were 0.78 when both spring + winter and spring only reference sets were used, whereas with FImpute they were 0.68 and 0.71, respectively. The accuracy of the imputation followed the same trend, being higher for Eagle + Minimac3, compared to FImpute, with the exception of the imputed spring lines from the spring reference set (0.84 vs 0.87; Supplementary Table S1a). A decline in the accuracy of imputation was observed for low-frequency SNPs in both imputation algorithms. This decline was less pronounced when using Eagle + Minimac3 (Supplementary Fig. S2). Based on these results we used Eagle + Minimac3 to impute all lines from 64,072 to 6 million SNPs.

A total of 585 individuals genotyped at 64,072 SNP were imputed to WGS using 153 individuals with 6 million SNPs as a reference set. Sporadic missing genotypes were imputed using Beagle^47^ in both datasets followed by phasing with Eagle, which in turn was followed by the actual imputation using Minimac^50^. Based on the Minimac results, we set a Rsqr (squared correlation between imputed and posterior genotypes) threshold of > 0.1, which dropped the final SNP density to 1,234,708 (Supplementary Table S1b). Supplementary Table S1b also shows that other slightly altered pipelines resulted in lower Rsqr values. Imputation accuracy and the proportion of correctly imputed SNP markers (accuracy) are presented in Supplementary Table S1b. Beagle + Eagle + Minimac was chosen to impute whole-genome sequence for GWAS.

**Figure 1:**
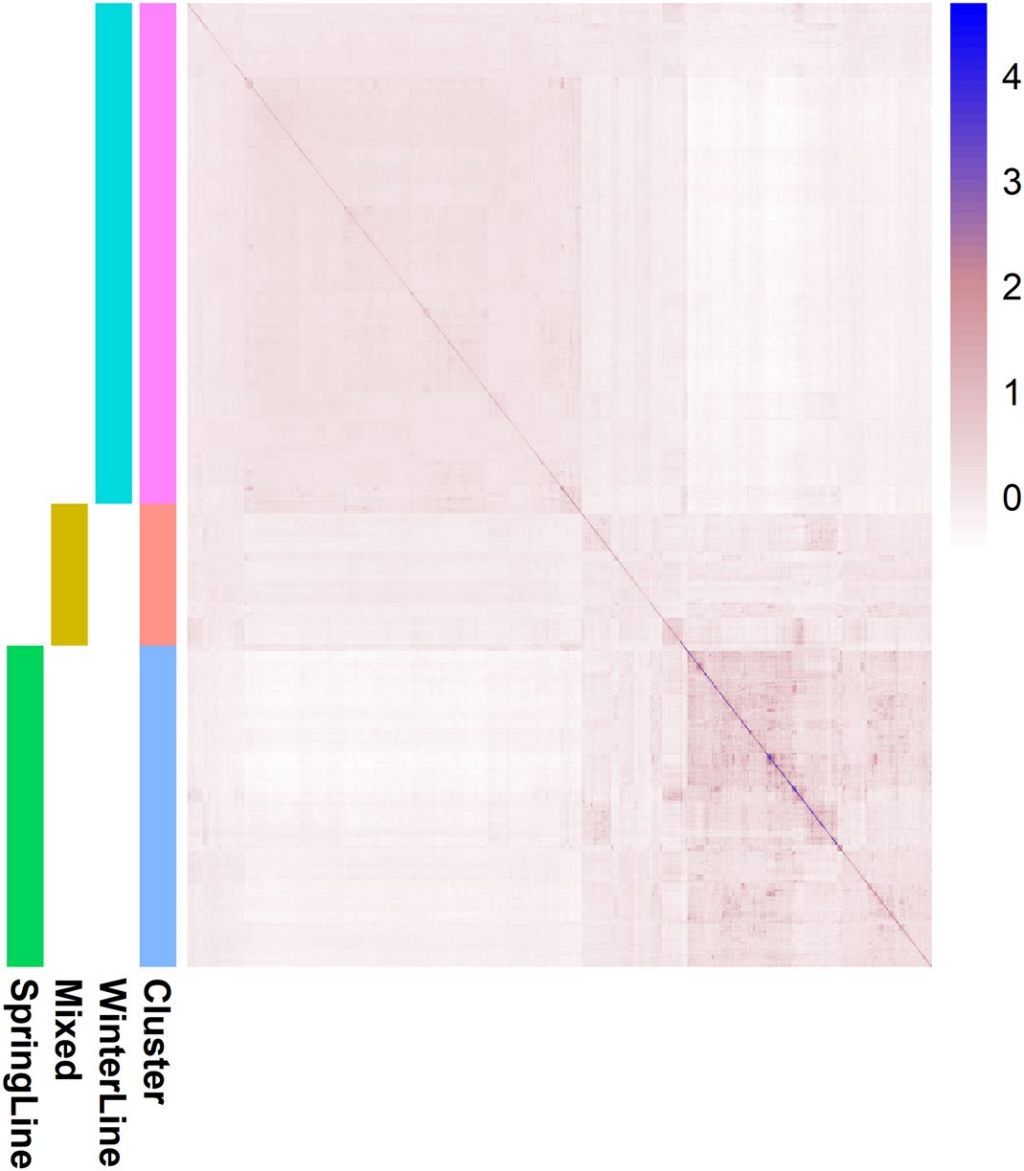
Heat map of the genomic relationship matrix for 585 diverse canola lines using the imputed 1,234,708 SNP markers. Mixed = spring canola lines with winter background; darker colour indicates greater relatedness. Figure produced in R3.6.

The genetic relatedness between 585 canola lines was investigated with a genomic relationship matrix as described in Yang et al.^55^. The population were clustered into two main categories (Fig. 1). The larger cluster defined the winter lines followed by the spring set. A few spring lines with winter background formed a separate cluster, confirming that these populations were originally derived from winter lines.

### Single trait genome-wide association study and false discovery rate

**Figure 2:**
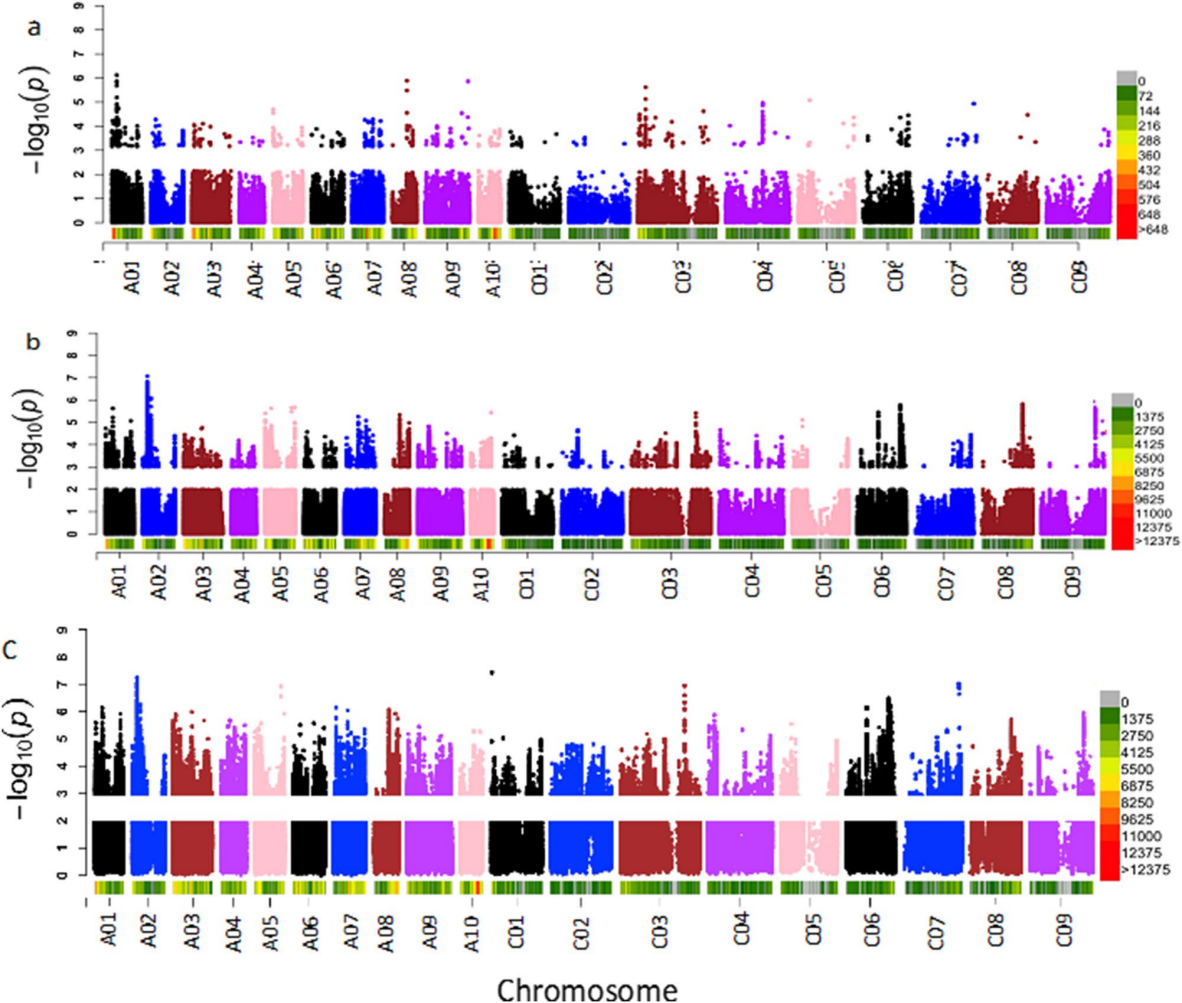
Increased power of WGS and meta-analysis of GWAS for internal infection as demonstrated by Manhattan plots for (**a**) transcriptomic genotyping-by-sequence (GBSt) at Wickliffe and (**b**) imputed whole-genome sequence (WGS) at Wickliffe and (**c**) multi-trait meta-analysis of GWAS for internal infection and survival at the two 2015 blackleg trials. The colour bar shows SNP density every 1Mbp. Figure produced in R3.6 using CMplot function (https://github.com/YinLiLin/R-CMplot**).**

**Figure 3:**
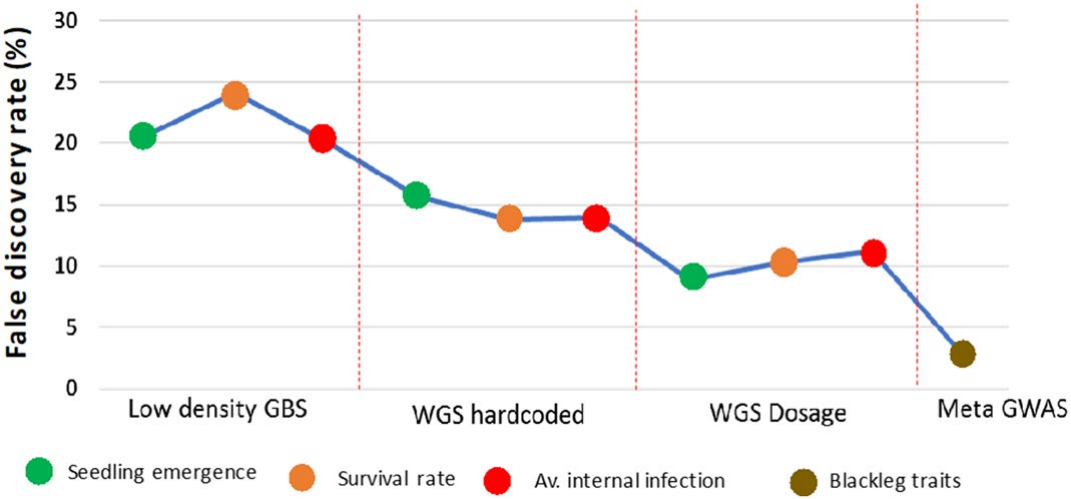
False discovery rate (at $P < 1 \times 10^{ - 4}$) for GWAS using GBSt dosage, WGS dosage, WGS hard coded genotypes based on blackleg disease prone site (Wickliffe) and meta-analysis for blackleg disease traits (internal infection and survival). Figure produced in R3.6.

Manhattan plots of single-trait GWAS using WGS and GBSt for emergence count, survival rate, and average internal infection at Wickliffe and Green Lake sites are shown in Supplementary Figs. S3abc, S4abc and S5 and QQ plots indicated that population structure was properly accounted for (Supplementary Fig. S6). Using WGS, a considerably larger number of significant SNPs than for the GBSt GWAS were detected at all P-value thresholds and for all traits (Table 2). In general, similar peaks were identified for GBSt and WGS, but the WGS GWAS revealed additional peaks (Fig. 2a vs b). Furthermore, we repeated the WGS GWAS with hardcoded genotypes (most probable genotype coded as 0, 1, and 2), which generally identified a similar number of significant SNP at $P \le 1 \times 10^{ - 4}$ and fewer significant SNP at $P \le 1 \times 10^{ - 5}$ with higher FDR. The general trend of power based on FDR was, therefore, WGS dosage > WGS hardcoded > GBSt dosage (Fig. 3).

**Table 2:** Comparison of GBSt versus WGS GWAS as well as two alternative ways to code genotypes (dosage and hardcoded) for emergence (EME), average internal infection (AvInf), and survival (Surv) at Wickliffe (WL) and Green Lake (GL) sites in 2015, where FDR is the false discovery rate.

**Traits**	Single trait GWAS using GBSt dosage genotypes				Single trait GWAS using WGS dosage genotypes				Single trait GWAS using WGS in 012 genotypes			
	No. of sig. SNPs and FDR at 4 p-values				No. of sig. SNPs and FDR at 4 p-values				No. of sig. SNPs and FDR at 4 p-values			
	p < 10^−3^	p < 10^−4^	p < 10^−5^	p < 10^−6^	p < 10^−3^	p < 10^−4^	p < 10^−5^	p < 10^−6^	p < 10^−3^	p < 10^−4^	p < 10^−5^	p < 10^−6^
EMEWL	437	31	11	0	5,942	1,396	269	4	5,525	787	104	3
FDR(%)	14.7	20.7	5.8	–	20.8	8.8	4.6	40.2	22.4	15.7	11.9	41.2
SurvWL	586	27	14	1	5,719	1,197	101	3	6,807	895	93	2
FDR(%)	10.9	23.7	4.6	6.4	21.6	10.3	12.2	41.2	18.1	13.8	12.1	61.7
AvInfWL	639	32	18	1	4,895	1,106	287	3	4,936	889	97	1
FDR(%)	10.1	20.1	3.6	6.4	25.2	11.2	4.3	41.2	25.1	13.9	12.7	123.5
EMEGL	414	38	4	0	5,259	954	220	1	5,641	801	12	2
FDR(%)	15.5	16.9	16.1	–	23.5	12.9	5.6	123.5	21.9	15.4	102.9	61.7
SurvGL	593	36	3	0	5,112	972	214	14	5,211	797	86	2
FDR(%)	10.9	17.8	21.4	–	24.2	12.7	5.8	8.8	23.8	15.5	14.4	61.7
AvInfGL	669	27	8	0	4,782	816	68	1	5,213	902	32	1
FDR(%)	10.1	23.7	8.1	–	25.8	15.1	18.2	123.5	23.7	13.7	38.6	123.5

### Meta-analysis of GWAS

**Figure 4:**
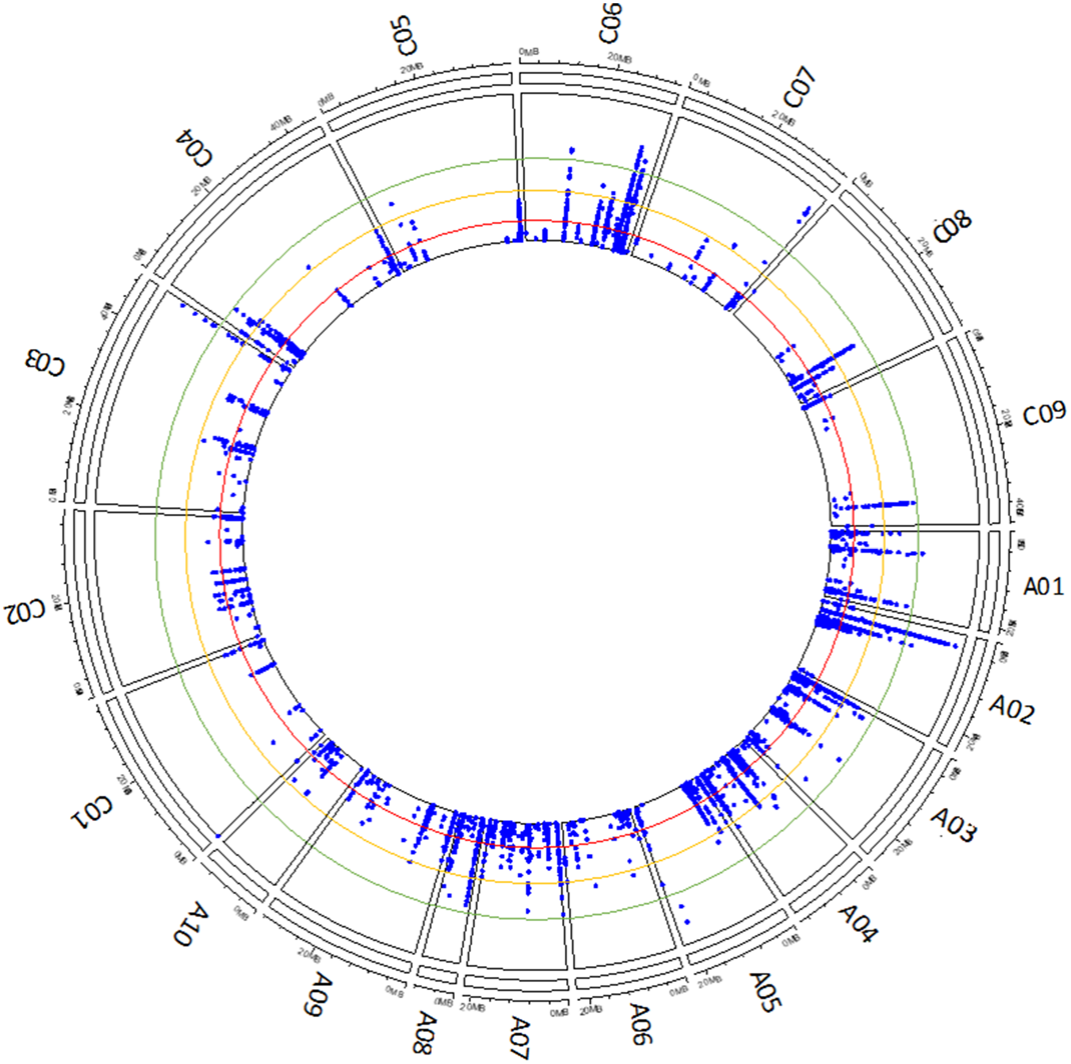
A circular plot showing potential candidate genomic region associated with blackleg traits in *Brassica napus* L. across A and C sub-genome at $P < 1 \times 10^{ - 3} ; 10^{-4};10^{ - 5} ;\;{\text{and}}\;10^{ - 6}$ thresholds shown as black, red, yellow and green lines, respectively. The top 50 significant SNPs across the regions are indicated in the circle. Figure produced in R3.6.

Two meta-analyses were carried out combining the single-trait WGS dosage GWAS to increase power and to identify pleiotropic loci. One combined internal infection and survival (blackleg related) phenotypes across the two sites in 2015 (Figs. 2c, 4), and the other combined all traits and sites in that year. For the blackleg related traits, 101 SNPs were significant ($P < 1 \times 10^{ - 6}$) with an FDR of 1.22%, which is a considerable improvement when compared to the single-trait WGS GWAS (Figs. 2c, 3; Tables 2, 3). Adding emergence GWAS into the meta-analysis increased the number of significant SNP, however, this trend stopped at $P < 1 \times 10^{ - 6}$ (Table 3). In addition, we compared a GWAS model that combined BLUEs from different sites to combining separate site GWAS using a meta-analysis (Table 4). The meta-analyses detected more significant SNP at all p-value thresholds.

**Table 3:** Meta-analysis for specific to blackleg traits and combined all-traits for the 2015 trials. FDR = False discovery rate.

	Meta-analysis for blackleg traits				Meta-analysis all-traits			
	No. of sig. SNPs and FDR at 4 p-values				No. of sig. SNPs and FDR at 4 p-values			
	p < 10^−3^	p < 10^−4^	p < 10^−5^	p < 10^−6^	p < 10^−3^	p < 10^−4^	p < 10^−5^	p < 10^−6^
No. of Sig SNP	28,192	4,488	674	101	31,195	5,758	1,019	107
FDR (%)	4.4%	2.8%	1.8%	1.2%	3.9%	2.1%	1.2%	1.2%

**Table 4:** Meta-analysis using a combined best linear unbiased estimates (BLUEs) from the Wickliffe and Green Lake 2015 trials.

Traits	Combined site GWAS model				Meta-analysis to combine sites			
	No. of sig. SNPs and FDR at 4 p-values				No. of sig. SNPs and FDR at 4 p-values			
	p < 10^−3^	p < 10^−4^	p < 10^−5^	p < 10^−6^	p < 10^−3^	p < 10^−4^	p < 10^−5^	p < 10^−6^
EME	9,210	1,279	20	3	11,917	1606	151	7
FDR(%)	13.4	9.6	61.7	41.2	10.4	7.7	8.2	17.6
Surv	8,480	1,205	60	2	9,982	1,343	264	12
FDR(%)	14.6	10.3	20.6	61.7	12.4	9.2	4.7	10.3
AvInf	10,010	1,376	90	2	13,122	1,486	103	9
FDR(%)	12.3	8.9	13.7	61.7	9.6	8.3	11.9	13.7

EME = emergence count, surv = survival rate, AvInf = Average internal infection, FDR = false discovery rate.

### Validation and comparison of results from different GWAS strategies

**Figure 5:**
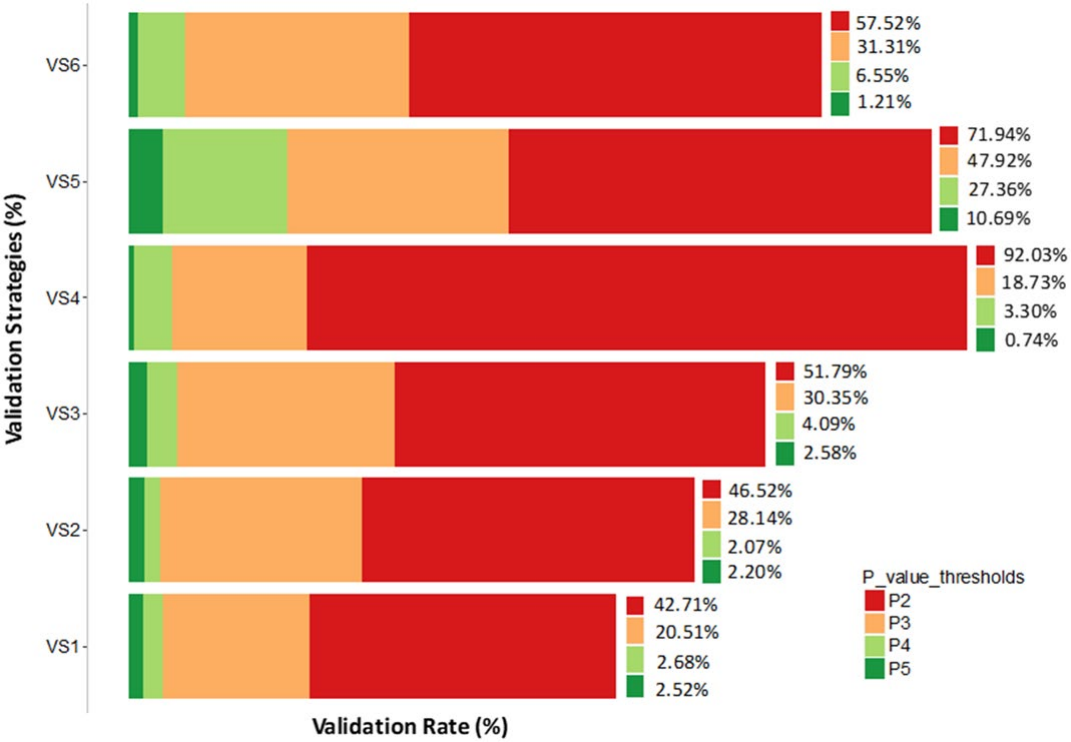
Mean validation rates (%) of different validation strategies across four P-value thresholds ($P < 1 \times 10^{ - 2}$; $P < 1 \times 10^{ - 3}$; $P < 1 \times 10^{ - 4} ; P < 1 \times 10^{ - 5}$). Input information in Supplementary Tables S1–S6. Validation strategies are **VS1**) single-GBSt-to-single-GBSt: single-trait GWAS 2015 in dosage GBSt in discovery set and single-trait GWAS in GBSt dosage validation in 2016 and 2017 **VS2**) single-012-to-single-012: WGS single-trait GWAS 2015 in 012 (integer) in a discovery set and WGS single-trait GWAS in 012 (integer) validation in 2016 and 2017 **VS3**) single-to-single: single-trait dosage GWAS 2015 in discovery set and single-trait dosage GWAS validation in 2016 and 2017 **VS4**) meta-to-single: meta-analysis GWAS of blackleg traits in 2015 and validation in single trait GWAS in 2016 and 2017 **VS5**) CombModel-to-single: Combined model sites per traits GWAS in 2015 and validation in single trait GWAS in 2016 and 2017. **VS6**) meta-AvInt-to-singleAvInt: meta-analysis for AvInt GWAS in 2015 and validation in single-trait AvInt GWAS in 2016 and 2017. Figure produced in R3.6.

We used the 2016 and 2017 datasets to validate and compare the different GWAS approaches. We implemented various validation strategies (VS) and quantified the most significant SNPs at $P < 1 \times 10^{ - 5}$ identified in several disovery sets: single-trait GWAS analysis using GBSt (VS1); single-trait GWAS hardcoded (VS2); WGS GWAS within locations; single-trait dosage (VS3); multi-trait meta-analysis for blackleg traits using dosage WGS genotypes (VS4); full linear mixed model to combine locations per trait (VS5); meta-analysis combining 2 locations per trait (VS6); and (Fig. 5; Supplementary Tables S2–S7).

Validation strategy 1 (VS1) involved a single-trait GWAS–GBSt dosage discovery set during 2015 at Wickliffe and Green Lake sites with validation in single-trait GWAS–GBSt dosage in 2016 and 2017 trials for EME and AvInf. We found that a smaller number of significant SNPs validated in VS1 versus VS2, which validated the single-trait GWAS–WGS hardcoded discovery set in single-trait GWAS–hardcoded in the same datasets (Fig. 5). We further extended the validation strategy (VS2) to discover and validate significant SNPs detected in single-trait GWAS–WGS dosage genotypes (VS3) and found VS3 to be superior in its validation rates at all p-value thresholds. Comparing VS2 and VS3 to VS1, we can conclude that imputed WGS GWAS were more powerful than GBSt GWAS in this dataset. A slightly different trend was observed when significant SNPs discovered in blackleg meta-analysis GWAS were validated using single-trait GWAS at 2016 and 2017 sites (VS4). Compared to VS1-3, we observed a higher validation rate for P < 10^−2^ and P < 10^−4^ in VS4, but a lower validation rate for P < 10^−3^ and P < 10^−5^. This shows that meta-analysis improved the overall validation rate, but when specifically looking at highly significant SNP in both the discovery and validation GWAS, it validated a lower proportion of SNPs. Validation strategies 5 (VS5) and 6 (VS6) tested whether it is better to combine data per trait with a multi-location GWAS model (and fitting location as fixed) versus combining locations with a meta-analysis of single location GWAS, respectively. The validation rates observed clearly indicated that combining locations in a full model (VS5) is preferable to simply performing a meta-analysis of single location GWAS (VS6), which is to some extent expected. It is notable that for P < 10^−3^, the location full GWAS model (VS5) performed better than the blackleg meta-analysis (VS4).

Validation rate (Vr) calculated as:

$${\text{Vr}} = { }\frac{{{\text{Number}}\,{\text{of}}\,{\text{significant }}\,{\text{NPs}}\,{\text{acheived}}\,{\text{in}}\,{\text{the}}\,{\text{validation}}\,{\text{at}}\,{\text{P}}\,{\text{value}}}}{{{\text{Number}}\,{\text{of}}\,{\text{significant}}\,{\text{SNPs}}\,{\text{in}}\,{\text{the}}\,{\text{discovery}}\,{\text{set}}\,{\text{at}}\,{\text{P}}\,{\text{value}}}}$$

### Pinpointing candidate genomic regions identified for resistance to *L. maculans*

The number of significant SNPs (at $P < 1 \times 10^{ - 4}$) in the single-trait GWAS analysis varied between blackleg traits and across experimental sites. In general, a higher number of significant SNPs were found at the Wickliffe site compared to Green Lake, while the number of discoveries for the traits within location was relatively similar.

The meta-analysis of blackleg GWAS discovered a larger number of additional SNPs when compared to the single trait GWAS. The most significant SNPs (P = 9.59E−07) were located on 3685 kb of chromosome A02 followed by ChrA08, ChrC03, and ChrC06 (Fig. 4, Supplementary Tables S8, S9 and S10). To identify potential candidate genes with putative resistance to *L. maculans*, the 674 significant SNPs obtained from meta-analysis were clustered into separate regions if the distance between SNP was more than 200 kb. A total of 79 potential regions were observed (Supplementary Tables S8 and S10). Furthermore, when we compared within the A and C sub-genomes, 51 genomic regions consisting of 344 significant SNPs were on the A sub-genome, while 330 SNP were within 28 regions on the C sub-genomes. The regions with the most significant SNPs were on chromosome C06 (157 SNP) followed by chromosome A02 (125 SNP) and A09 (64 SNP) and A03 (55 SNP). No significant SNP (P < 1 × 10^−5^) were detected on C02, and very few significant SNP were observed on C01 and C05 (Supplementary Table S8, Fig. 4). Overall, many of the significant results detected in our study shared similar regions with known Rlm genes and QTLs found in linkage mapping populations.

### Candidate gene mining for blackleg disease traits using BLAST

*B. napus* SNP effect annotations revealed that most significant SNP were intergenic, followed by intronic, synonymous, non-synonymous, stop gained and lost coding functions (Supplementary Tables S9 and S10). We performed a BLASTn against *Arabidopsis thaliana* for *B. napus* gene sequences within 30 kb up and downstream of the 674 significantly associated SNP $\left( {P < 1 \times 10^{ - 5} } \right)$ from the blackleg meta-analysis. The BLAST cut-off e-value threshold was set as e−7. The percentage of identical matches, number of nucleic mismatches, expected e-value and bit score were estimated. We searched the BLASTn results with the following search terms; such as disease, resistance, fungus, pathogen, pathogenesis, stem canker, stress, bacteria and abnormality. Based on this we found potential candidate genes with known biotic and abiotic resistant QTLs associated with *A. thaliana* accounting for 17 out of the 79 genomic regions, with functions that positively related to disease resistance and plant stress.

## Discussion

We have reported GWAS results for seedling emergence and blackleg traits (internal infection and survival) and we have validated and compared the results from different GWAS strategies across multiple locations and years. We conducted single-trait and multi-trait meta-analysis GWAS using WGS and GBSt in a diverse set of canola lines. The imputation to WGS was reasonably accurate and the imputation pipeline using Eagle + Minimac3 seemed to outperform FImpute. WGS-based GWAS were more powerful than using GBSt as indicated by the number of significant SNPs, the FDR, and the validation rates in additional years of field trial data. Meta-analysis identified more significant SNP than single trait GWAS with a similar FDR, but a smaller percentage of SNP were validated across years from the meta-analysis. GWAS using dosage were more powerful than hardcoded genotypes, where power was judged by the validation rates and FDR. Combining locations with multi-trait GWAS model increased validation rates substantially over combining single-trait GWAS results with a meta-analysis. The blackleg GWAS meta-analysis revealed 79 major genome regions (containing SNP P < 10^−4^) putatively involved in resistance to *L. maculans*. The *B. napus* genes in the vicinity of these significant SNP were mapped with BLASTn against the *A. thaliana* genome, which revealed several genes with putative functions related to disease resistance.

### Factors affecting the power of GWAS

The imputation of WGS into diverse reference population lines enabled an increase in the sample size for the GWAS. We have shown that the accuracy of imputation was > 0.71. This level of accuracy is similar to what has been previously achieved in other crops^54^. However, it is substantially less than in human and other mammals^56–58^. Aside from the differences in the size of the WGS reference set, there are several possible reasons for this discrepancy. Mammals are diploid and their reference genome assemblies (particularly human) tend to have fewer errors than crop assemblies. Many crops are polyploid, often with significant homology between the sub-genomes in allopolyploids (e.g. canola) and this brings challenges to infer genotype dosage in autoploids. This adds complexity to inferring assemblies and to mapping reads to unique genome locations, which, in turn, leads to mapping errors and false positive SNPs. We have performed stringent quality control in our dataset in an attempt to reduce the number of erroneous SNPs and genotypes. Nevertheless, a large proportion of our imputed SNPs achieved only a low Minimac Rsq, which seems to indicate a reasonably large false positive SNP or genotyping error rate. Imputation accuracy could possibly be further improved with an improved *B. napus* genome assembly, a denser GBSt dataset coupled with greater fold coverage, a greater focus on sub-genome specific SNP, a larger WGS reference set, and more stringent quality control.

We have mitigated the effect of SNP and genotype uncertainty in two ways. First, we have imposed a Minimac Rsqr threshold of 0.1, which corresponds to an approximate empirical imputation accuracy of 0.4. Using only SNP exceeding this threshold is helpful in reducing the false positive rate and reducing the multiple testing problem by only interrogating better imputed SNPs. Secondly, we have investigated using both hardcoded (0,1,2) and dosage genotypes in our GWAS analysis to evaluate its effect on power. Dosage imputed WGS genotypes were found to be better at pinpointing causal mutations in dairy cattle^57^. Indeed, we found that dosage genotypes detected more significant SNPs with a lower FDR and with higher validation rates than hardcoded genotypes indicating that they improved the GWAS power. The improved power is thought to come from the modelling of uncertainty in the imputed genotype. If there is a lot of uncertainty the genotype will be closer to the heterozygote genotype, thereby reducing their influence in the analysis and potentially leading to fewer false positive associations.

The largest increase in GWAS power was observed from using WGS imputed sequences rather than sequences obtained by GBSt alone. This phenomenon has been demonstrated in a number of species, including *A. thaliana*^59^; rice^60^ and in crop species in general, see a review by Edwards and Batley^61^. In our study, imputation to WGS accomplished two goals. Firstly, it increased the number of SNPs and therefore increased the linkage disequilibrium between markers to increase their association with causal loci. Secondly, WGS imputation augmented the dataset with intergenic markers, which can harbor regulatory variants. The increase in power has led to additional discoveries of regions involved in blackleg disease resistance. This initial resource of WGS individuals can be expanded and improved to make future GWAS more effective. Additionally, it remains to be tested whether WGS will improve genomic prediction.

The majority of GWAS are fitted as single traits and environments. We have combined single trait GWAS using a meta-analysis approach^42^. An increase in power was observed previously from meta-analyses, whose aim is to discover loci that affect multiple traits^42,62–64^. Similarly, the advantages of large-scale meta-analysis for genetic mapping in plant were reviewed in several studies^59,65,66^ and suggested that meta-analysis provides prospects for the identification of genes relevant to trait improvement. In our case combining internal infection and survival from the 2015 trials in a meta-analysis substantially increased the number of significant SNPs and reduced the FDR. Validating the meta-analysis SNPs in the internal infection GWAS collected from the 2016 and 2017 trials revealed a higher validation rate for higher p-value levels or a lower validation rate for lower p-values compared to single trait GWAS (Fig. 5, Supplementary Fig. S6).

Blackleg fungal populations are highly dynamic and multiple populations exist in the Victorian environment^31^. We attempted to mitigate this by growing the two blackleg disease nurseries in 2015 on stubble from the same cultivar. Nevertheless, disease pressure could have varied across the two 2015 sites. Meta-analysis may be less effective than single trait GWAS for highly significant, possibly fungal population specific, loci, while at the same time it could be more effective for less significant loci that may be components of quantitative resistance. The environment of the 2016 and 2017 trials used for validation was clearly managed differently (i.e. treatments with fungicide) to the 2015 trials. Overall, this, along with the smaller samples size of later trials, would have depressed the validation rates. However, as all GWAS strategies were assessed on the same datasets the comparisons are expected to be fair. If the original phenotype and genotype data are available, it is preferable to fit an appropriate within trait full model with a fixed effect of location to combine data rather than meta-analysis. In our case, the full model was more successful at validation as p values became more stringent, indicating a lower rate of false positives. Future work could investigate a multi-trait GWAS to a multi-trait meta-analysis of single trait GWAS.

There are additional ways to validate SNPs identified in discovery sets, for example using partially known lines from the previous year datasets, as was shown in maize^66^ and rice^67^ , and using different lines from independent nursery, as shown in bread wheat^68^. Alternatively, a cross-validation approach could be used for validation^40,69^. Furthermore linkage mapping approaches^31,70^ in bi-parental populations have been used to validate significant markers identified in GWAS hits in canola and wheat.

### Putative candidate regions and genes for resistance to *L. maculans*

Previous studies have identified major genes and genomic regions conferring resistance to *L. maculans* using primarily bi-parental populations and found many such regions are clustered on chromosome A07 and A10, see two reviews^71,72^. In addition, single-trait GWAS have, to date, identified > 334 putative loci for blackleg (*L. maculans*) disease resistance^31,38,73^.

We have arbitrarily defined significant genomic regions as separate if the distance between SNP was more than 200 kb. The size of region capturing potentially the same causative mutation is directly related to the amount of linkage disequilibrium (LD) in our population. Malmberg et al.^74^ determined in the same population that LD dropped to 0.2 at approximately 200 kb distance between SNPs, a level which has been confirmed in other studies^25,75^. This resulted in a total of 79 potential genomic regions across the A and C sub-genomes, of which 52 were new. While the remaining 27 of these putative regions were near previously published Rlm genes and quantitative QTL, further follow-up is required to confirm that our signals are indeed for known Rlm genes. Without a physical location for many published QTL it is often difficult to confirm whether our significant SNPs overlapped with previous linkage study results. When comparing cM from linkage groups and our physical positions our estimates of overlap were only approximations.

Four regions were identified on A01 of which one is near the Rlm12 region and potentially other QTL^23–25,31^. On A02, all four regions overlapped with previous results and included the LepR1 resistance gene^21,24,25^. Not surprisingly, these regions included many of our most significant SNP, hence other studies have also found them. Chromosome A03 harboured eight potential regions of which only one had been previously identified^24,25^. Several of the new regions contained only one or two SNP, that meet the significance threshold, indicating that the signal in for these regions was less strong. Seven regions were found on A04 with one previously published^24,25^. One region on A05 confirmed a previous report^25^ and four were new. Our GWAS detected LepR4 and other previously published QTL^24,25,76^ as well as two new regions on A06. All seven regions on A07 overlapped with published resistance genes (Rlm1, Rlm3, Rlm4, Rlm9, LmFr1, LMR1 and LEM1) as well as quantitative QTL^11–19,21,23^. Five regions were found on A08, of which one overlapped with Rlm5 and several QTL^23–25,77^, and four regions were new. One region of A09 overlapped with several QTL^24,25^ and three were novel. Two significant regions on A10 collocated with Rlm2, LepR3, BLMR1, BLMR2, and LepR2^16,49,76^. Our observed signals in these two regions are likely associated to quantitative resistance rather than known major resistance genes, because Rlm2/LepR3 and LepR2 are defeated genes in the Australian context.

A substantial number of regions potentially harbouring blackleg disease resistance were found on the C sub-genome. Chromosome C01 contained one new and one previously known region^23^. No significant SNP were found on C02. Two and seven new regions were detected on C03 and C04, respectively, where one region on C04 was published^25^. C05 harboured one new region and nine new regions were found on C06 (one new region contained 80 significant SNP), of which one was previously discovered^23^. Rlm6, which was introgressed from *B. juncea* and is known to be present in Australian germplasm, was confirmed on C07 along with three new regions. Two regions were discovered on C08^24,25^, where one was new. Finally, C09 contained a new putative blackleg resistance region.

Assembly annotation is a crucial component to understanding and interpreting GWAS results. Many of our GWAS regions fell into the intergenic class, this is similar to findings in other species including mammals. It has also been observed in plants and algae^78^. Intergenic variants may have potential roles in transcriptional or translational regulation. The annotation of regulatory regions is complex and requires techniques that provide information outside of genic regions. Some examples of such assays are MNase, ChIP sequencing and HiC that provide information on open and closed chromatin regions and DNA folding^11–19,38^.

Due to the significant homology of genes, information can be shared across species. We further compared genomic regions detected in *A. thaliana* with previously known *L. maculans* resistant canola genes and found that a gene near one of our significant SNP and overlapping with Rlm2^49^ had high homology with the AT1G01170.2 gene localized in *A. thaliana* Chr1-74105–74443, which is thought to have a role in stress responsive conditions. As Rlm2 is thought to be defeated in Australia, we speculate that our signal captured other resistance loci in the same region. Similarly, a gene near the locus for the *B. juncea*-introgressed Rlm5^77^ blackleg resistant gene aligned to the AT1G10090.1 gene in Chr1 of *A. thaliana*. This gene is thought to be involved in stress responsive dehydration. The Victorian pre-breeding lines in our dataset do contain *B. juncea* introgressions. Moreover, we showed that candidate resistance genes were mapped in the vicinity of known R genes in *B. napus* genome and *A. thaliana* genes responsible for various stressors. This has demonstrated the importance of additional genomic resources in *Brassica* species such as TAIR (https://www.arabidopsis.org/) and Genoscope (https://www.genoscope.cns.fr/brassicanapus/) to enhance the discovery of new genes using related model plants. Several studies in other species confirm the benefits of BLAST to align putative candidate genes detected in GWAS analysis in wild wheat, tree species^79^ and canola^31^; *B. juncea*^80^; *B. oleracea*^81^. Overall, understanding the nature of genetic inheritance of a trait and genomic regions underlying association with blackleg traits will provide a new insight into the genetic architecture of the trait and accelerates the development of resistant cultivars.

## Material and methods

### Plant materials and trait measurements

We used data from 585 canola lines (391 winter and 194 spring types) grown at Wickliffe (37.665839° S, 142.754126° E) and Green Lake (36.768420° S, 142.264679° E), Victoria, Australia during 2015 growing season, previously described in Fikere et al.^32^ (Supplementary Table S11). Similarly, we conducted additional agronomic field trials under irrigated (Horsham irrigated 2016 and 2017) and rain-fed (Mininera 2016 and Horsham rain-fed 2017) conditions during the 2016 and 2017 growing seasons. Data from these agronomic trials (seedling emergence and internal infection) were used for the validation step of this study. The 2015 trial was a single-row disease nursery for blackleg sown in the canola stubble retained from the previous year’s crop (ATR-Gem). The trial was designed in AGROBASE using a randomized complete block design with a check variety grid every 10th row (var. Trigold). Two replicates were used at each location. The agronomic trials conducted in 2016 and 2017 were randomized incomplete block designs with 2–3 reps in each location, sown in 7.5 m^2^ plots. The following phenotypes were recorded from the disease nursery: emergence count (number of plants emerged 6 weeks after sowing), blackleg traits such as adult plant survival rate and average internal infection of the stem at maturity. The agronomic trials were protected from blackleg disease twice, by using fungicide at sowing (impact-in-furrow) and a treatment at the 6–8 leaf stage with Prosaro 420 SC foliar fungicide. Thus, the blackleg management protocol was different in years 2016 and 2017, and these trials were only used for validation purposes. To determine the internal infection of the stem, we employed a modified method of the “Blackleg Canker Test” used in the Australian National Variety Trials (Grain Research Development Corporation, personal communications on NVT “The Protocols”, version 4, 9th July 2014). Briefly, in the 2015 trial, a total of 20 plants per row were sampled from a minimum of 7 randomly chosen positions (i.e. 3 consecutive plants from 7 widely spaced positions within each row, including any dead or lodged plants). For the 2016 and 2017 trials, 5 consecutive plants in a middle row in each plot were sampled to generally survey for any level of blackleg in these fungicide protected trials. The sampled plants were then cut with secateurs at the crown and the cross section of the stem was examined. The area of the stem infected by *L. maculans* was recorded as a percentage of affected area of the total stem cross-section, as described in the Spring Blackleg Management Guide^82^. Plot values were used in analyses and, for average internal infection, this was the mean value of multiple stems in each plot.

### Phenotype processing

Phenotypes were spatially adjusted with autocorrelation error (i.e. AR1 x AR1) models for field condition variability to generate Best Linear Unbiased Estimates (BLUEs) using ASReml^83^, as described in^32,84^. The model was as follows:

$$y_{ijk} = \mu + g_{i} + r_{j} + c_{k} + e_{ijk}$$

where $y_{ijk}$ is the phenotype, $\mu$ is the overall mean, $g_{i}$ is the fixed effect for variety $i$, $r_{j}$ is the random effect for row $j$, $c_{k}$ is the random effect for column $k$ fit as a spline, and $e_{ijk}$ is the residual. The variance due to lines (Vg) was estimated using the above model with $g_{i}$ fitted as random and broad-sense heritabilities were calculated as Vg/(Vg + Ve/nrep), where Ve was the residual variance and nrep was the number of replicates per line (nrep = 2 in all trials)^85^.

### Transcriptome genotyping-by-sequencing (GBSt)

A total of 585 Spring and Winter type canola lines were genotyped using the protocol described in Malmberg et al.^86^. Briefly, mRNA was extracted from leaf tissue (Dynabeads: Life Technologies) and used for library preparation for RNA sequencing (SureSelect: Agilent Technologies). Circa 3 million reads were generated per sample using either an Illumina HiSeq3000 or a NextSeq500. The resulting sequence data was adaptor and quality trimmed before aligning to the Darmor-*bzh* whole genome reference^87^ using the Tophat2 algorithm^88^ and SNP were identified with SAMtools mpileup^89^.

### Whole genome sequencing (WGS)

Whole genome sequence data from 153 samples covering the diversity of the 585 GBSt lines described in^74^ were re-analyzed. The sequencing protocols were fully described in^86^. Briefly, whole genome libraries were generated for all samples and sequenced on an Illumina HiSeq3000 aiming for 10 × read depth coverage per sample. The resulting sequence data was adaptor and quality trimmed before aligning to the Darmor-*bzh* whole genome reference^87^ using the BWA mem algorithm^90^, and SNP were identified with SAMtools mpileup^89^.

### Quality control

We implemented stringent quality control thresholds on 6 million WGS variants based on minor allele frequency (MAF > 0.1), missing rate per SNPs and missing rate per sample (< 50%), genotype read depth (DP > 5), heterozygosity rate per SNP (Hetero > 0.4) followed by imputation accuracy from Minimac R-square (Rsqr > 0.1). Finally, genotypes were checked for SNP duplications and any duplicates were removed keeping one of the set in the refined data. The final dataset was checked for their MAF > 0.01, giving a total of 153 lines and 1,234,708 million SNPs for subsequent analysis.

### Sequence imputation

We assessed the imputation accuracy of two imputation pipelines: FImpute (no pedigree option^48^ and Eagle V2.3^49^ followed by Minimac3 V2.0.1^50^ in the set of 153 whole-genome sequenced lines via fivefold cross validation. Locations not present in the GBSt assay were masked in validation lines and imputed from the WGS data. The accuracy of imputation was assessed as the correlation and concordance of imputed and observed sequence genotypes. Additionally, we investigated whether it is beneficial to combine winter and spring lines for imputation or consider them separately. To do so, imputation was conducted within spring lines, within winter lines separately and using spring lines as a validation and winter lines as reference set and vice versa in a tenfold cross validation. After this evaluation, the Eagle2.3 Minimac3 pipeline was used for imputing the 585 lines from GBSt density to WGS using the entire set of 153 lines as the reference set. Minimac3 provides genotypes in full dosage format (i.e. real numbers ranging from 0 to 2) and in hardcoded genotype format (i.e. coded as 0, 1, and 2, for homozygous reference, heterozygous, and homozygous alternative respectively) for the imputed sequence variants. We investigated the effect of these formats on the power of GWAS.

### Genome-wide association analysis

We used EMMAX (Effective Mixed Model Association eXpedited) to perform GWAS^91^ analysing one SNP at a time with a linear mixed model. EMMAX makes the simplifying and time-saving assumption that any given SNP’s effect on the trait is typically small and therefore only estimates the model variance components once per analysis to account for population structure. EMMAX estimates the variance components with the REML model

$${\mathbf{y}} = {\mathbf{Wb}} + {\mathbf{Zg}} + {\mathbf{e}}$$

where **y** was a vector BLUEs, **W** and **Z** were incidence matrices, **b** was a vector of fixed effects including intercept and seasonal type (winter or spring), **g** (${\mathbf{g}} \sim N\user2{ }\left( {0, \sigma_{g}^{2} {\mathbf{G}}} \right))$ and **e** (${\mathbf{e}}\user2{ }\sim \user2{ }N\left( {0,{ }\sigma_{e}^{2} {\mathbf{I}}} \right))$ were vectors of additive genetic effects and residuals, respectively, and $\sigma_{g}^{2}$ was the additive genetic and $\sigma_{e}^{2}$ was the residual variance. **G** was the genomic relationship matrix calculated following Yang et al.^55^. An F-test is then calculated per SNP using the estimates of the polygenic and residual terms from the variance component step. GWAS were run per site and year. An additional model of combined BLUEs per trait and location was fitted as a fixed effect was run to investigate the effect of combining sites within year.

### Meta-analysis of single-trait GWAS

The meta-analysis used the SNP effects from single-trait GWAS, as described in Bolormaa et al.^42^. A $\chi^{2}$ test statistic was calculated as follows:

$$\chi^{2} = t_{i}^{^{\prime}} V^{ - 1} t_{i}$$

where $t_{i}$ was number of traits *k* × 1 vector of the signed t-values of SNP_*i*_ effects, i.e., beta/se, for the *k* traits; *t*_*i*_′ was the transpose *t*_*i*_ (1 × *k*); and V^−1^ was the inverse of the *k* × *k* correlation matrix, where the correlations were calculated for all signed t-values per pair of traits. The $\chi^{2}$ value of each SNP were examined for significance based on a $\chi^{2}$ distribution with *k* degrees of freedom. False discovery rates were calculated as $FDR = \frac{P * T}{A} \times 100,$ where P is the p-value tested, T is the total number of SNP tested and A is the number of SNP that were significant at the p-value tested. In addition, meta-analyses were used to combine single-trait GWAS across the two locations and to combine the traits survival rate and internal infection from the 2015 trials.

### Validation of GWAS in two additional years of field trial data

GWAS were analysed in several different ways. Our aim was to validate which method resulted in the most power. A priori it is difficult to determine which method is more powerful. One way to judge the utility of an approach, aside from FDR, is how many of the significant SNP remain significant in a new dataset. In this study, we used emergence and internal infection values from the four agronomical field trials in two additional seasons (2016 and 2017) for validation. We performed the following validation strategies (VS): (**VS1**) single-GBSt-to-single-GBSt: single-trait GWAS 2015 in dosage GBSt in discovery set and single-trait GWAS in GBSt dosage validation in 2016 and 2017; (**VS2**) single-012-to-single-012: WGS single-trait GWAS 2015 in 012 (integer) in a discovery set and WGS single-trait GWAS in 012 (integer) validation in 2016 and 2017; (**VS3**) single-to-single: WGS single-trait dosage GWAS 2015 in discovery set and WGS single-trait dosage GWAS validation in 2016 and 2017; (**VS4**) meta-to-single: WGS meta-analysis GWAS of blackleg traits in 2015 and validation in WGS single trait GWAS in 2016 and 2017; (**VS5**) CombModel-to-single: WGS combined model sites per traits GWAS in 2015 and validation in WGS single trait GWAS in 2016 and 2017; (**VS6**) meta-AvInt-to-singleAvInt: WGS meta-analysis for AvInt GWAS in 2015 and validation in WGS single-trait AvInt GWAS in 2016 and 2017. Significance thresholds were chosen based on false discovery rates.

### Performing BLAST for *B. napus* L. GWAS significant SNP against *A. thaliana*

In addition, to gain an understanding of the underlying gene functions, we used BLASTn (Basic Local Alignment Search Tool) analysis against the *Arabdophsis thaliana* sequence database (https://www.arabidopsis.org/). Gene sequences within 30 kb up or downstream of significant SNPs detected in meta-analysis at the 10^−5^ p-value were included in the BLASTn analysis. BLAST matches to multiple loci with the same top identity metrics were removed. Prediction of functional variant annotation of an individual SNPs was performed using SnpEff^92^ using the *B. napus* Darmor-*bzh* genome annotation file v5 (gff3) and the whole genome reference sequence^87^. Details of the annotation procedures is provided in Malmberg et al.^74^. Finally, potential genes from the meta-analysis were used to define the regions of interest that contain potential candidate genes.

## Supplementary information

Supplementary file 1.

Supplementary file 2.

Supplementary file 3.

## Abbreviations

AFLP: Amplified fragment length polymorphism
BLAST: Basic local alignment score tool
BLUEs: Best linear unbiased estimates
DH: Doubled haploid
EMMAX: Effective mixed model association expedited
FDR: False discovery rate
GWAS: Genome-wide association study
LD: Linkage disequilibrium
MAF: Minor allele frequency
QC: Quality control
SNP: Single nucleotide polymorphism
GBSt: Transcriptome genotype-by-sequencing
VS: Validation strategy
WGS: Whole-genome sequence

## References

[1] USDA. https://ipad.fas.usda.gov/rssiws/al/global_cropprod.aspx (accessed on 26 June, 2018). (2016).

[2] Hwang S-F (2016). Blackleg (*Leptosphaeria maculans*) severity and yield loss in canola in Alberta, Canada. Plants.

[3] Aubertot JN, Pinochet X, Dore T (2004). The effects of sowing date and nitrogen availability during vegetative stages on *Leptosphaeria maculans* development on winter oilseed rape. Crop Prot..

[4] Sprague S (2006). Major gene resistance in *Brassica napus* (oilseed rape) is overcome by changes in virulence in populations of *Leptosphaeria maculans* in France and Australia. Eur. J. Plant Physiol..

[5] Cowling W (2007). Genetic diversity in Australian canola and implications for crop breeding for changing future environments. Field Crops Res..

[6] Van de Wouw AP (2010). Evolution of linked avirulence effectors in *Leptosphaeria maculans* is affected by genomic environment and exposure to resistance genes in host plants. PLoS Pathog..

[7] Delourme R (2006). Major gene and polygenic resistance to *Leptosphaeria maculans* in oilseed rape (*Brassica napus*). Eur. J. Plant Pathol..

[8] Huang Y-J, Qi A, King GJ, Fitt BDL (2014). Assessing quantitative resistance against *Leptosphaeria maculans* (Phoma Stem Canker) in *Brassica napus* (Oilseed Rape) in young plants. PLoS ONE.

[9] Sosnowski MR, Scott ES, Ramsey MD (2004). Infection of Australian canola cultivars (*Brassica napus*) by *Leptosphaeria maculans* is influenced by cultivar and environmental conditions. Australas. Plant Pathol..

[10] Sosnowski MR, Scott ES, Ramsey MD (2005). Temperature, wetness period and inoculum concentration influence infection of canola (*Brassica napus*) by pycnidiospores of *Leptosphaeria maculans*. Australas. Plant Pathol..

[11] Dion Y, Gugel RK, Rakow GFW, Séguin-Swartz G, Landry BS (1995). RFLP mapping of resistance to the blackleg disease [causal agent, *Leptosphaeria maculans* (Desm.) Ces. et de Not.] in canola (*Brassica napus* L.). Theor. Appl. Genet..

[12] Ferreira ME, Satagopan J, Yandell BS, Williams PH, Osborn TC (1995). Mapping loci controlling vernalization requirement and flowering time in Brassica napus. Theoretical and Applied Genetics.

[13] Mayerhofer R, Good AG, Bansal VK, Thiagarajah MR, Stringam GR (1997). Molecular mapping of resistance to *Leptosphaeria maculans* in Australian cultivars of *Brassica napus*. Genome.

[14] Delourme R (2004). A cluster of major specific resistance genes to *Leptosphaeria maculans* in *Brassica napus*. Phytopathology.

[15] Yu F, Lydiate D, Rimmer S (2005). Identification of two novel genes for blackleg resistance in *Brassica napus*. Theor. Appl. Genet..

[16] Yu F, Lydiate DJ, Rimmer SR (2007). Identification and mapping of a third blackleg resistance locus in *Brassica napus* derived from *B. rapa* subsp. sylvestris. Genome.

[17] Parlange F (2009). *Leptosphaeria maculans* avirulence gene AvrLm4-7 confers a dual recognition specificity by the Rlm4 and Rlm7 resistance genes of oilseed rape, and circumvents Rlm4-mediated recognition through a single amino acid change. Mol. Microbiol..

[18] Kaur S (2009). Genetic map construction and QTL mapping of resistance to blackleg (*Leptosphaeria maculans*) disease in Australian canola (*Brassica napus* L.) cultivars. Theor. Appl. Genet..

[19] Pilet ML, Delourme R, Foisset N, Renard M (1998). Identification of loci contributing to quantitative field resistance to blackleg disease, causal agent *Leptosphaeria maculans* (Desm.) Ces et de Not., in Winter rapeseed (*Brassica napus* L.). Theor. Appl. Genet..

[20] Jestin C (2011). Association mapping of quantitative resistance for *Leptosphaeria maculans* in oilseed rape (*Brassica napus* L.). Mol. Breed..

[21] Ghanbarnia K (2012). Genetic mapping of the *Leptosphaeria maculans* avirulence gene corresponding to the LepR1 resistance gene of *Brassica napus*. Theor. Appl. Genet..

[22] Larkan NJ (2014). Co-localisation of the blackleg resistance genes Rlm2 and LepR3 on *Brassica napus* chromosome A10. BMC Plant Biol..

[23] Larkan NJ (2016). Multi-environment QTL studies suggest a role for cysteine-rich protein kinase genes in quantitative resistance to blackleg disease in *Brassica napus*. BMC Plant Biol..

[24] Huang YJ (2016). Identification of environmentally stable QTL for resistance against *Leptosphaeria maculans* in oilseed rape (*Brassica napus*). Theor. Appl. Genet..

[25] Kumar V (2018). Multi-year linkage and association mapping confirm the high number of genomic regions involved in oilseed rape quantitative resistance to blackleg. Theor. Appl. Genet..

[26] Alamery S (2018). Genome-wide identification and comparative analysis of NBS-LRR resistance genes in *Brassica napus*. Crop Pasture Sci..

[27] Bayer PE, Golicz AA, Scheben A, Batley J, Edwards D (2020). Plant pan-genomes are the new reference. Nat. Plants.

[28] Hurgobin B, Edwards D (2017). SNP discovery using a pangenome: has the single reference approach become obsolete?. Biology (Basel).

[29] Hurgobin B (2017). Homoeologous exchange is a major cause of gene presence/absence variation in the amphidiploid *Brassica napus*. Plant Biotechnol. J..

[30] Hu ZY, Huang SM, Sun MY, Wang HZ, Hua W (2012). Development and application of single nucleotide polymorphism markers in the polyploid *Brassica napus* by 454 sequencing of expressed sequence tags. Plant Breed..

[31] Raman H (2016). Genome-wide association study identifies new loci for resistance to *Leptosphaeria maculans* in canola. Front. Plant Sci..

[32] Fikere M (2018). Genomic prediction using prior quantitative trait loci information reveals a large reservoir of underutilised blackleg resistance in diverse canola (*Brassica napus* L.) lines. Plant Genome.

[33] Yano K (2016). Genome-wide association study using whole-genome sequencing rapidly identifies new genes influencing agronomic traits in rice. Nat. Genet..

[34] Hu G (2017). Genome-wide association study identified multiple genetic loci on chilling resistance during germination in maize. Sci. Rep..

[35] Sun L (2015). GmHs1–1, encoding a calcineurin-like protein, controls hard-seededness in soybean. Nat. Genet..

[36] Badouin H (2017). The sunflower genome provides insights into oil metabolism, flowering and Asterid evolution. Nature.

[37] Thambugala D (2013). Genetic variation of six desaturase genes in flax and their impact on fatty acid composition. Theor. Appl. Genet..

[38] Fomeju BF, Falentin C, Lassalle G, Manzanares-Dauleux MJ, Delourme R (2014). Homoeologous duplicated regions are involved in quantitative resistance of *Brassica napus* to stem canker. BMC Genom..

[39] Wei L (2016). Genome-wide association analysis and differential expression analysis of resistance to *Sclerotinia stem* rot in *Brassica napus*. Plant Biotechnol J.

[40] Bolormaa S (2016). Detailed phenotyping identifies genes with pleiotropic effects on body composition. BMC Genom..

[41] Xiang RD, MacLeod IM, Bolormaa S, Goddard ME (2017). Genome-wide comparative analyses of correlated and uncorrelated phenotypes identify major pleiotropic variants in dairy cattle. Sci. Rep..

[42] Bolormaa S (2014). A multi-trait, meta-analysis for detecting pleiotropic polymorphisms for stature, fatness and reproduction in beef cattle. PLoS Genet..

[43] Trampush JW (2017). GWAS meta-analysis reveals novel loci and genetic correlates for general cognitive function: a report from the COGENT consortium. Mol. Psychiatry.

[44] Kilpeläinen TO (2016). Genome-wide meta-analysis uncovers novel loci influencing circulating leptin levels. Nat. Commun..

[45] Willer CJ (2008). Newly identified loci that influence lipid concentrations and risk of coronary artery disease. Nat. Genet..

[46] Howie BN, Donnelly P, Marchini J (2009). A flexible and accurate genotype imputation method for the next generation of genome-wide association studies. PLOS Genet..

[47] Browning SR, Browning BL (2007). Rapid and accurate haplotype phasing and missing-data inference for whole-genome association studies by use of localized haplotype clustering. Am. J. Hum. Genet..

[48] Sargolzaei M, Chesnais JP, Schenkel FS (2014). A new approach for efficient genotype imputation using information from relatives. BMC Genom..

[49] Loh P-R (2016). Reference-based phasing using the Haplotype Reference Consortium panel. Nat. Genet..

[50] Howie B, Fuchsberger C, Stephens M, Marchini J, Abecasis GR (2012). Fast and accurate genotype imputation in genome-wide association studies through pre-phasing. Nat. Genet..

[51] Chan AW, Hamblin MT, Jannink J-L (2016). Evaluating imputation algorithms for low-depth genotyping-by-sequencing (GBS) data. PLoS ONE.

[52] Xavier A, Muir WM, Rainey KM (2016). Impact of imputation methods on the amount of genetic variation captured by a single-nucleotide polymorphism panel in soybeans. BMC Bioinform..

[53] Torkamaneh D, Belzile F (2015). Scanning and filling: ultra-dense SNP genotyping combining genotyping-by-sequencing, SNP array and whole-genome resequencing data. PLoS ONE.

[54] Shi F (2017). Exome sequence genotype imputation in globally diverse hexaploid wheat accessions. Theor. Appl. Genet..

[55] Yang J (2010). Common SNPs explain a large proportion of the heritability for human height. Nat. Genet..

[56] Browning BL, Browning SR (2016). Genotype imputation with millions of reference samples. Am. J. Hum. Genet..

[57] Pausch H (2017). Evaluation of the accuracy of imputed sequence variant genotypes and their utility for causal variant detection in cattle. Genet. Select. Evol..

[58] Bouwman AC (2018). Meta-analysis of genome-wide association studies for cattle stature identifies common genes that regulate body size in mammals. Nat. Genet..

[59] Korte A, Farlow A (2013). The advantages and limitations of trait analysis with GWAS: a review. Plant Methods.

[60] Misra G (2017). Whole genome sequencing-based association study to unravel genetic architecture of cooked grain width and length traits in rice. Sci. Rep..

[61] Edwards D, Batley J (2009). Plant genome sequencing: applications for crop improvement. Plant Biotechnol. J..

[62] Evangelou E, Ioannidis JPA (2013). Meta-analysis methods for genome-wide association studies and beyond. Nat. Rev. Genet..

[63] Thompson JR, Attia J, Minelli C (2011). The meta-analysis of genome-wide association studies. Brief Bioinform..

[64] Willer CJ, Li Y, Abecasis GR (2010). METAL: fast and efficient meta-analysis of genomewide association scans. Bioinformatics.

[65] Xu Y, Li P, Yang Z, Xu C (2017). Genetic mapping of quantitative trait loci in crops. Crop J..

[66] Thiemann A (2014). Genome-wide meta-analysis of maize heterosis reveals the potential role of additive gene expression at pericentromeric loci. BMC Plant Biol..

[67] Lu Y (2015). Systems genetic validation of the SNP-metabolite association in rice via metabolite-pathway-based phenome-wide association scans. Front Plant Sci..

[68] Mourad AMI (2018). Genome-wide association study for identification and validation of novel SNP markers for Sr6 stem rust resistance gene in bread wheat. Front. Plant Sci..

[69] Cao K (2016). Genome-wide association study of 12 agronomic traits in peach. Nat. Commun..

[70] Pasam RK (2017). Detection and validation of genomic regions associated with resistance to rust diseases in a worldwide hexaploid wheat landrace collection using BayesR and mixed linear model approaches. Theor. Appl. Genet..

[71] Van De Wouw AP, Marcroft SJ, Howlett BJ (2016). Blackleg disease of canola in Australia. Crop Pasture Sci..

[72] Raman, H., Raman, R. & Larkan, N. Genetic dissection of blackleg resistance loci in rapeseed (*Brassica napus* L.) *InTech*, 86–119. 10.5772/53611 (2013).

[73] Raman H (2014). Genome-wide delineation of natural variation for pod shatter resistance in *Brassica napus*. PLoS ONE.

[74] Malmberg MM, Shi F, Spangenberg GC, Daetwyler HD, Cogan NOI (2018). Diversity and genome analysis of Australian and global oilseed *Brassica napus* L. germplasm using transcriptomics and whole genome re-sequencing. Front. Plant Sci..

[75] Ecke W, Clemens R, Honsdorf N, Becker HC (2010). Extent and structure of linkage disequilibrium in canola quality winter rapeseed (*Brassica napus* L.). Theor Appl Genet.

[76] Yu F, Gugel RK, Kutcher HR, Peng G, Rimmer SR (2013). Identification and mapping of a novel blackleg resistance locus LepR4 in the progenies from *Brassica napus* × *B. rapa* subsp. sylvestris. Theor. Appl. Genet..

[77] Balesdent MH, Attard A, Kühn ML, Rouxel T (2002). New avirulence genes in the phytopathogenic fungus *Leptosphaeria maculans*. Phytopathology.

[78] Zhao Z (2014). Genome-wide analysis of tandem repeats in plants and green algae. G Genes Genom. Genet..

[79] Ćalić I (2017). Genome-wide association study identifies a major gene for beech bark disease resistance in American beech (*Fagus grandifolia* Ehrh.). BMC Genom..

[80] Yang J (2016). The genome sequence of allopolyploid *Brassica juncea* and analysis of differential homoeolog gene expression influencing selection. Nat. Genet..

[81] Golicz AA (2016). The pangenome of an agronomically important crop plant *Brassica oleracea*. Nat. Commun..

[82] GRDC. Spring Blackleg Management Guide: https://www.nvtonline.com.au/wp-content/uploads/2015/12/Blackleg-Management-Guide-Spring-2015-All-site-info. Accessed 15 May 2017. (2015).

[83] Gilmour, A. R., Cullis, B. R., Gogel, B. J., Welham, S. J. & Thompson, R. ASReml User Guide Release 2.0. VSN International Ltd, Hemel Hempstead, HP1 1ES, UK (2005).

[84] BurgueñoWeigel JDlCGK, Crossa J (2012). Genomic prediction of breeding values when modelling genotype-environment interaction using pedigree and dense molecular markers. Crop Sci..

[85] 87Holland James, B., Nyquist Wyman, E. & Cervantes-Martínez Cuauhtemoc, T. Estimating and Interpreting Heritability for Plant Breeding. *In Plant Breeding Reviews, J. Janick (Ed.).* **22**, 9–112, 10.1002/9780470650202.ch2 (2002).

[86] Malmberg MM (2017). Genotyping-by-sequencing through transcriptomics: implementation in a range of crop species with varying reproductive habits and ploidy levels. Plant Biotechnol. J..

[87] Chalhoub B (2014). Early allopolyploid evolution in the post-Neolithic *Brassica napus* oilseed genome. Science.

[88] Kim D (2013). TopHat2: accurate alignment of transcriptomes in the presence of insertions, deletions and gene fusions. Genome Biol..

[89] Li H (2009). The Sequence Alignment/Map format and SAMtools. Bioinformatics.

[90] Li, H. Aligning sequence reads, clone sequences and assembly contigs with BWA-MEM. arXiv preprint arXiv:1303.3997 (2013).

[91] Kang HM (2010). Variance component model to account for sample structure in genome-wide association studies. Nat. Genet..

[92] Cingolani P (2012). A program for annotating and predicting the effects of single nucleotide polymorphisms, SnpEff. Fly.

