# Supplementary material for: Meta-analysis of GWAS in canola blackleg (Leptosphaeria maculans) disease traits demonstrates increased power from imputed whole-genome sequence
Source: Sci Rep. 2020 Aug 31;10:14300. doi: 10.1038/s41598-020-71274-6 (PMC7459325; doi:10.1038/s41598-020-71274-6)
Supplement: Supplementary file 1 [file 41598_2020_71274_MOESM1_ESM.docx]

Meta-Analysis of GWAS in Canola Blackleg (*Leptosphaeria maculans*) Disease Traits Demonstrates Increased Power from Imputed Whole-Genome Sequence

M. Fikere^1,2,3^, D. M. Barbulescu^4^, M.M. Malmberg^1,2^, G.C. Spangenberg^2,1^, N.O.I Cogan^2,1^, H.D. Daetwyler^2,1✝^

^1^School of Applied Systems Biology, La Trobe University, Bundoora 3086, VIC, Australia

^2^Agriculture Victoria, AgriBio, Centre for AgriBioscience, Bundoora 3083, VIC, Australia

^3^ Queensland Alliance for Agriculture and Food Innovation (QAAFI), The University of Queensland, 4072 Brisbane QLD - Australia

^4^Agriculture Victoria, Grains Innovation Park, Horsham 3401, VIC, Australia

^✝^Corresponding author: [hans.daetwyler@agriculture.vic.gov.au](mailto:hans.daetwyler@agriculture.vic.gov.au)

Telephone: +61(0) 3 9032 7037

**List of Supplementary Figure Legends**

**Figure 1S.** Correlation of BLUEs between traits and their distribution across field trials. EME = emergence count, surv = survival rate, Avint = Average internal infection, WL = Wickliffe, GL = Green Lake, MI = Mininera, HrI16 = Horsham irrigated 2016, HrI17 = Horsham irrigated 2017, Hr17 = Horsham rain-fed. Figure produced in R3.6.

**Figure S2.** The correlation between observed and imputed genotypes as a function of the minor allele frequency in canola lines. Figure produced in R3.6.

**Figure S3a**. Manhattan plot based on Whole-genome sequence variants dataset for emergence count (a), survival rate (b) and average internal infection (c) at Wickliffe site. Figure produced in R3.6 using CMplot function ([https://github.com/YinLiLin/R-CMplot](https://urldefense.proofpoint.com/v2/url?u=https-3A__github.com_YinLiLin_R-2DCMplot&d=DwMGaQ&c=JnBkUqWXzx2bz-3a05d47Q&r=1IJkAaTQ_1vFhZoul-i6eHlbAvPwETYDJSI3v8KU38Zn92T2QYvyanexkV0nffDD&m=MDpBSgNNkDIg2XPIaoVJxR5JYFkO7hMDHNz1hsy7J1g&s=Cvo3PTtHYQ4RRRJHAt88yxJVuE12RsdgHtyYyd7yVeQ&e=)**).**

**Figure S3b**. Manhattan plot based on Whole-genome sequence variants dataset for emergence count (a), survival rate (b) and average internal infection (c) at Green Lake site. Figure produced in R3.6 using CMplot function ([https://github.com/YinLiLin/R-CMplot](https://urldefense.proofpoint.com/v2/url?u=https-3A__github.com_YinLiLin_R-2DCMplot&d=DwMGaQ&c=JnBkUqWXzx2bz-3a05d47Q&r=1IJkAaTQ_1vFhZoul-i6eHlbAvPwETYDJSI3v8KU38Zn92T2QYvyanexkV0nffDD&m=MDpBSgNNkDIg2XPIaoVJxR5JYFkO7hMDHNz1hsy7J1g&s=Cvo3PTtHYQ4RRRJHAt88yxJVuE12RsdgHtyYyd7yVeQ&e=)**).**

**Figure S4a**. Manhattan plot based on GBS dataset for emergence count (a), survival rate (b) and average internal infection (c) at Wickliffe site. Figure produced in R3.6 using CMplot function ([https://github.com/YinLiLin/R-CMplot](https://urldefense.proofpoint.com/v2/url?u=https-3A__github.com_YinLiLin_R-2DCMplot&d=DwMGaQ&c=JnBkUqWXzx2bz-3a05d47Q&r=1IJkAaTQ_1vFhZoul-i6eHlbAvPwETYDJSI3v8KU38Zn92T2QYvyanexkV0nffDD&m=MDpBSgNNkDIg2XPIaoVJxR5JYFkO7hMDHNz1hsy7J1g&s=Cvo3PTtHYQ4RRRJHAt88yxJVuE12RsdgHtyYyd7yVeQ&e=)**).**

**Figure S4b**. Manhattan plot based on GBS dataset for emergence count (a), survival rate (b) and average internal infection (c) at Green Lake site. Figure produced in R3.6 using CMplot function ([https://github.com/YinLiLin/R-CMplot](https://urldefense.proofpoint.com/v2/url?u=https-3A__github.com_YinLiLin_R-2DCMplot&d=DwMGaQ&c=JnBkUqWXzx2bz-3a05d47Q&r=1IJkAaTQ_1vFhZoul-i6eHlbAvPwETYDJSI3v8KU38Zn92T2QYvyanexkV0nffDD&m=MDpBSgNNkDIg2XPIaoVJxR5JYFkO7hMDHNz1hsy7J1g&s=Cvo3PTtHYQ4RRRJHAt88yxJVuE12RsdgHtyYyd7yVeQ&e=)**).**

**Figure S5.** Manhattan plot based on Whole genome sequence variants dataset for emergence count (a), survival rate (b) and average internal infection (c) for combined sites. Figure produced in R3.6 using CMplot function ([https://github.com/YinLiLin/R-CMplot](https://urldefense.proofpoint.com/v2/url?u=https-3A__github.com_YinLiLin_R-2DCMplot&d=DwMGaQ&c=JnBkUqWXzx2bz-3a05d47Q&r=1IJkAaTQ_1vFhZoul-i6eHlbAvPwETYDJSI3v8KU38Zn92T2QYvyanexkV0nffDD&m=MDpBSgNNkDIg2XPIaoVJxR5JYFkO7hMDHNz1hsy7J1g&s=Cvo3PTtHYQ4RRRJHAt88yxJVuE12RsdgHtyYyd7yVeQ&e=)**)**

**Figure S6**. The quantile-quantile plot in column representing Manhattan plots results in Figure 2, S3a, S3b, S4a, and S4b

**List of Supplementary Table Legends**

**Table S1a.** Imputation scenarios between sub-populations (spring and winter lines) using as reference (Ref) and validation (Val) sets based on 6 million whole-genome sequence variants.

**Table S1b.** Number of SNPs retained at several Minimac Rsqr thresholds across imputation pipelines. Beagle + Eagle + Minimac as chosen to impute whole-genome sequence for GWAS.

**Table S2**. Significant lead SNPs (at ${P<1 x 10}^{-5}$) detected in a meta-analysis for blackleg traits as a discovery set and single-trait GWAS for internal infection as a validated set tested under rain-fed and irrigated conditions.

**Table S3**. Single traits in Discovery vs single trait in validation (2016 - 2017).

**Table S4**. Significant lead SNPs (at ${P<1 x 10}^{-5}$) detected in combined BLUEs (locations) are validated using additional in-field trial as a validation set

**Table S5**. Significant lead SNPs (at ${P<1 x 10}^{-5}$) detected for average internal infection in combined traits in meta-analysis GWAS (discovery set) are validated using additional in-field trial as a validation set.

**Table S6**. Significant lead SNPs (at ${P<1 x 10}^{-5}$) detected in WGS in a single-trait analysis in integer genotype format are validated using single-trait analysis based on integer format WGS.

**Table S7**. Significant lead SNPs (at ${P<1 x 10}^{-5}$) detected in GBSt – dosage in a single-trait analysis are validated using single-trait GBSt – dosage format.

**Table S8**. List of known resistance genes, QTLs and new genomic regions associated with blackleg disease in canola.

**Table S9**. List of SNP effect annotation (Effect Classes count) across 19 Brassica napus L. chromosome.

**Table S10.** List of genes and genomic regions detected in both A annd C Brassica napus L. sub-genomes. Detail information on SNP annotation, gene onthology term and known genes along with refereces are provided

**Table S11**. List of accessions used in study and passport data, including Rlm gene presence where known.

**SUPPLEMENTARY FIGURES AND TABLES**

**Meta-Analysis of GWAS in Canola Blackleg (*Leptosphaeria maculans*) Disease Traits Demonstrates Increased Power from Imputed Whole-Genome Sequence**

M. Fikere^1,2,3^, D. Barbulescu^4^, M.M. Malmberg^1,2^, G.C. Spangenberg^2,1^, N.O.I Cogan^2,1^, H.D. Daetwyler^2,1^

Figure S1. Correlation of BLUEs between traits and their distribution across field trials. EME = emergence count, surv = survival rate, Avint = Average internal infection, WL = Wickliffe, GL = Green Lake, MI = Mininera, HrI16 = Horsham irrigated 2016, HrI17 = Horsham irrigated 2017, Hr17 = Horsham rain-fed

Figure S2. The correlation between observed and imputed genotypes as a function of the minor allele frequency in canola lines.


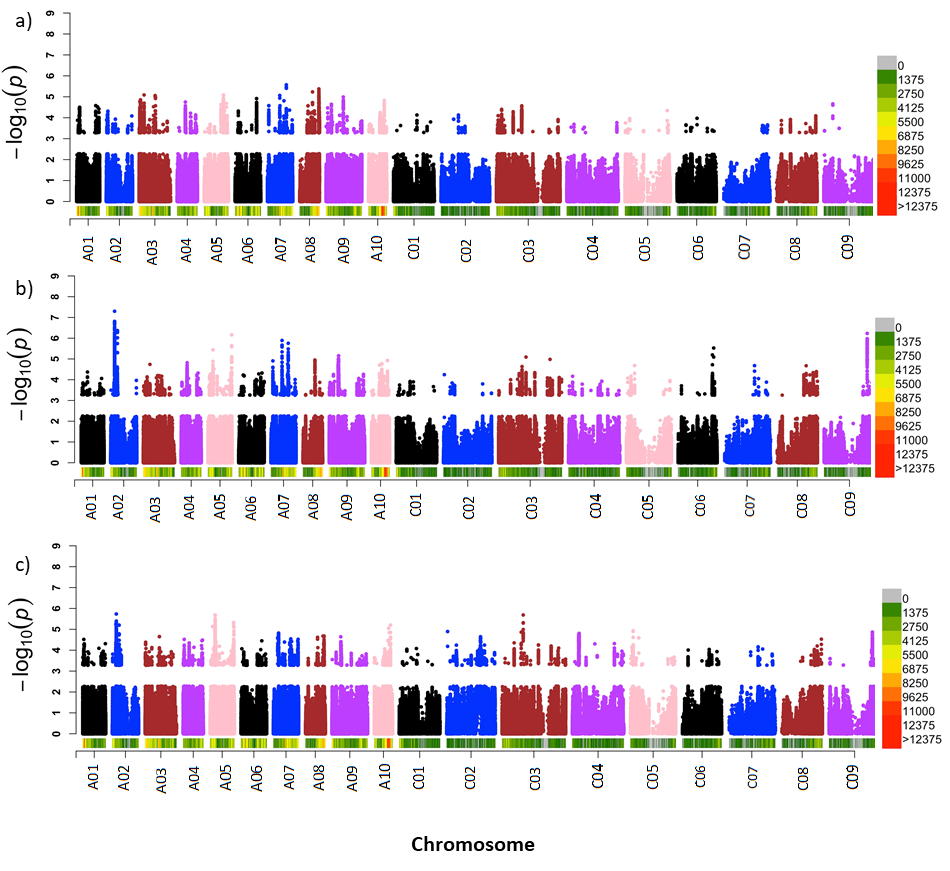


Figure S3a. Manhattan plot based on Whole-genome sequence variants dataset for emergence count (a), survival rate (b) and average internal infection (c) at Wickliffe site.

Figure S3b. Manhattan plot based on Whole-genome sequence variants dataset for emergence count (a), survival rate (b) and average internal infection (c) at Green Lake site.

Figure S4a. Manhattan plot based on GBS dataset for emergence count (a), survival rate (b) and average internal infection (c) at Wickliffe site.

Figure S4b. Manhattan plot based on GBS dataset for emergence count (a), survival rate (b) and average internal infection (c) at Green Lake site.


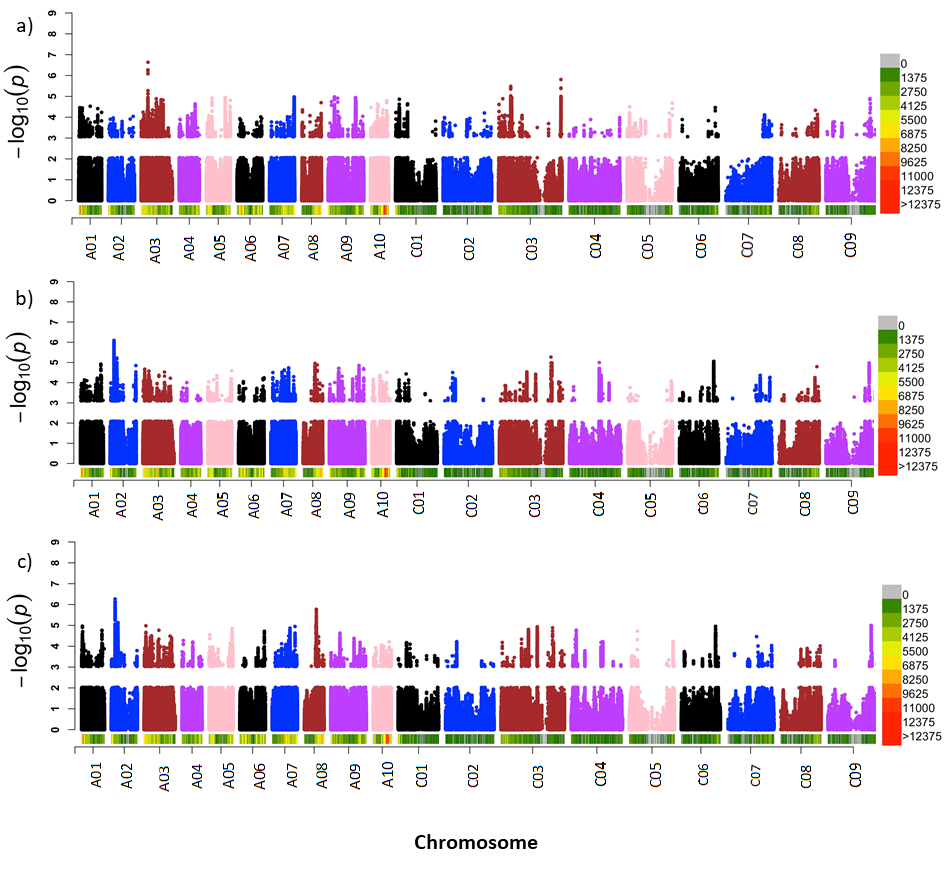


Figure S5. Manhattan plot based on whole genome sequence variants dataset for emergence count (a), survival rate (b) and average internal infection (c) for combined sites.


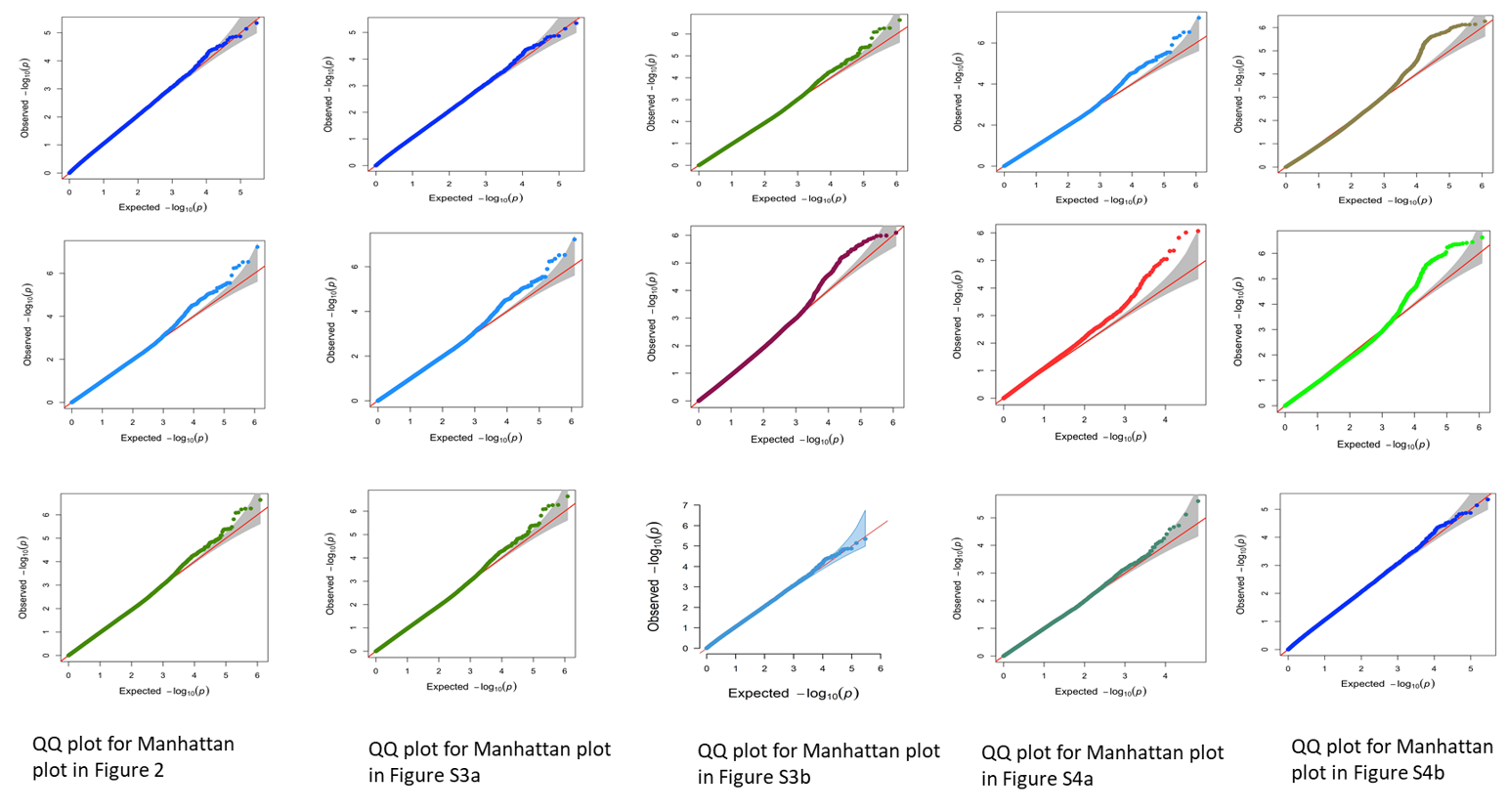


Figure S6. The quantile-quantile plot in column representing Manhattan plots results in Figure 2, S3a, S3b, S4a, and S4b

Table S1a. Imputation scenarios between sub-populations (spring and winter lines) using as reference (Ref) and validation (Val) sets based on 6 million whole-genome sequence variants

| Scenario | | FImpute | | | Eagle + Minimac3 | | |
| --- | --- | --- | --- | --- | --- | --- | --- |
| Val. | Ref. | Accuracy | Correlation | Accuracy | | Correlation |  |
| spring | spring + winter | 0.802 | 0.68 | 0.84 | | 0.78 |  |
| winter | winter + spring | 0.809 | 0.67 | 0.82 | | 0.71 |  |
| spring | spring | 0.869 | 0.71 | 0.84 | | 0.78 |  |
| winter | winter | 0.785 | 0.65 | 0.81 | | 0.73 |  |

Table S1b. Number of SNPs retained at several Minimac Rsqr thresholds across imputation pipelines. Beagle + Eagle + Minimac as chosen to impute whole-genome sequence for GWAS.

| Thresholds | Beagle + Eagle + Minimac | Eagle + Minimac | Beagle + Minimac |
| --- | --- | --- | --- |
| Rsqr Threshold | No. of SNPs retained | No. of SNPs retained | No. of SNPs retained |
| Initial | 6,683,199 | 6,683,199 | 6,683,199 |
| >=0.1 | 1,234,708 | 1,042,276 | 1,164,415 |
| >0.2 | 888,710 | 710,905 | 833,220 |
| >0.3 | 671,663 | 510,795 | 625,824 |
| >0.4 | 508,235 | 368,229 | 472,210 |
| >0.5 | 377,646 | 259,517 | 348,961 |

Table S2. Significant lead SNPs (at ${P<1 x 10}^{-5}$) detected in a meta-analysis for blackleg traits as a discovery set and single-trait GWAS for internal infection as a validated set tested under rain-fed and irrigated conditions

| Location in Validation | Traits | Number of SNPs in the Discovery set  at P < 1 x 10^-5^ | No. of significant SNPs in the validation set (${at-log}_{10}(p)$ | | | |
| --- | --- | --- | --- | --- | --- | --- |
|  |  |  | p<10^-2^ | p<10^-3^ | p<10^-4^ | p<10^-5^ |
| Mininera 2016 | Average internal infection | 674 | 670 | 130 | 32 | 7 |
|  | FDR(%) |  | 1.01 | 0.52 | 0.21 | 0.01 |
| Horsham irrigated 2016 | Average internal infection | 674 | 620 | 164 | 24 | 5 |
|  | FDR(%) |  | 1.08 | 0.41 | 0.28 | 0.13 |
| Horsham rain-fed 2017 | Average internal infection | 674 | 613 | 113 | 18 | 6 |
|  | FDR(%) |  | 1.10 | 0.60 | 0.37 | 0.11 |
| Horsham -irrigated 2017 | Average internal infection | 674 | 578 | 98 | 15 | 2 |
|  | FDR(%) |  | 1.17 | 0.69 | 0.45 | 0.34 |

Table S3. Single traits in Discovery vs single trait in validation (2016 - 2017)

| Location in Validation | Traits/sites | Number of SNPs in the Discovery set  at P < 1 x 10^-5^ | No. of significant SNPs in the validation set (${at-log}_{10}(p)$ | | | |
| --- | --- | --- | --- | --- | --- | --- |
|  |  |  | p<10^-2^ | p<10^-3^ | p<10^-4^ | p<10^-5^ |
|  | Wickliffe site 2015 |  |  |  |  |  |
| Mininera 2016 | Average internal infection | 287 | 98 | 16 | 3 | 1 |
|  | FDR (%) |  | 2.93 | 1.79 | 0.96 | 0.29 |
|  | Emergence score | 269 | 269 | 235 | 7 | 1 |
|  | FDR(%) |  | 2.51 | 0.29 | 0.96 | 0.67 |
| Horsham irrigated 2016 | Average internal infection | 287 | 56 | 51 | 6 | 1 |
|  | FDR(%) |  | 5.13 | 0.56 | 0.48 | 0.29 |
|  | Emergence score | 269 | 269 | 218 | 6 | 2 |
|  | FDR(%) |  | 2.51 | 0.31 | 1.12 | 0.17 |
| Horsham rain-fed 2017 | Average internal infection | 287 | 54 | 15 | 4 | 1 |
|  | FDR(%) |  | 5.315 | 1.913 | 0.718 | 0.287 |
|  | Emergence score | 269 | 208 | 36 | 17 | 3 |
|  | FDR(%) |  | 3.24 | 1.87 | 0.40 | 0.22 |
| Horsham –irrigated 2017 | Average internal infection | 287 | 69 | 19 | 7 | 3 |
|  | FDR(%) |  | 4.159 | 1.511 | 0.410 | 0.096 |
|  | Emergence score | 269 | 239 | 107 | 11 | 3 |
|  | FDR(%) |  | 2.82 | 0.63 | 0.61 | 0.22 |
|  | Green Lake site 2015 | |  |  |  |  |
| Mininera 2016 | Average internal infection | 68 | 36 | 11 | 2 | 1 |
|  | FDR(%) |  | 1.889 | 0.618 | 0.340 | 0.07 |
|  | Emergence score | 20 | 13 | 9 | 1 | - |
|  | FDR(%) |  | 0.14 | 0.03 | 0.02 | - |
| Horsham irrigated 2016 | Average internal infection | 68 | 27 | 14 | 2 | 1 |
|  | FDR(%) |  | 2.52 | 0.49 | 0.68 | 0.07 |
|  | Emergence score | 20 | 10 | 7 | 1 | 1 |
|  | FDR(%) |  | 0.20 | 0.03 | 0.02 | 0.001 |
| Horsham rain-fed 2017 | Average internal infection | 68 | 14 | 7 | 2 | 1 |
|  | FDR(%) |  | 4.86 | 0.97 | 0.34 | 0.07 |
|  | Emergence score | 20 | 14 | 10 | 2 | 1 |
|  | FDR(%) |  | 0.14 | 0.02 | 0.01 | 0.001 |
| Horsham irrigated 2017 | Average internal infection | 68 | 12 | 8 | 3 | 1 |
|  | FDR(%) |  | 5.667 | 0.85 | 0.223 | 0.07 |
|  | Emergence score | 20 | 10 | 8 | 2 | 1 |
|  | FDR(%) |  | 0.14 | 0.03 | 0.01 | 0.001 |

Table S4. Significant lead SNPs (at ${P<1 x 10}^{-5}$) detected in combined BLUEs (locations) are validated using additional in-field trial as a validation set

|  | Combined BLUEs (locations) | | | | | |
| --- | --- | --- | --- | --- | --- | --- |
| Location in Validation | Traits | Number of SNPs in the Discovery set  at P < 1 x 10^-5^ | No. of significant SNPs in the validation set (${at-log}_{10}(p)$ | | | |
|  |  |  | p<10^-2^ | p<10^-3^ | p<10^-4^ | p<10^-5^ |
| Mininera | Average internal infection | 90 | 75 | 47 | 16 | 6 |
|  | FDR(%) |  | 1.2 | 0.19 | 0.06 | 0.02 |
|  | Emergence score | 20 | 15 | 10 | 6 | 3 |
|  | FDR(%) |  | 1.333 | 0.20 | 0.03 | 0.01 |
| Horsham irrigated 2016 | Average internal infection | 90 | 65 | 37 | 15 | 8 |
|  | FDR(%) |  | 1.384 | 0.24 | 0.06 | 0.01 |
|  | Emergence score | 20 | 14 | 11 | 7 | 4 |
|  | FDR(%) |  | 1.428 | 0.182 | 0.029 | 0.005 |
| Horsham rain-fed | Average internal infection | 90 | 61 | 38 | 14 | 4 |
|  | FDR(%) |  | 1.475 | 0.24 | 0.05 | 0.02 |
|  | Emergence score | 20 | 15 | 12 | 9 | 3 |
|  | FDR(%) |  | 1.333 | 0.17 | 0.02 | 0.01 |
| Horsham -irrigated 2017 | Average internal infection | 90 | 56 | 25 | 17 | 5 |
|  | FDR(%) |  | 1.607 | 0.36 | 0.05 | 0.02 |
|  | Emergence score | 20 | 14 | 11 | 8 | 2 |
|  | FDR(%) |  | 1.428 | 0.18 | 0.03 | 0.01 |

Table S5. Significant lead SNPs (at ${P<1 x 10}^{-5}$) detected for average internal infection in combined traits in meta-analysis GWAS (discovery set) are validated using additional in-field trial as a validation set

|  | **Combined traits in Meta-analysis** | | | | | |
| --- | --- | --- | --- | --- | --- | --- |
| Location in Validation | **Traits** | Number of SNPs in the Discovery set  at P < 1 x 10^-5^ | No. of significant SNPs in the validation set (${at-log}_{10}(p)$ | | | |
|  |  |  | p<10^-2^ | p<10^-3^ | p<10^-4^ | p<10^-5^ |
| Mininera | Average internal infection | 103 | 67 | 35 | 10 | 1 |
|  | FDR |  | 1.537 | 0.271 | 0.103 | 0.103 |
| Horsham irrigated 2016 | Average internal infection | 103 | 58 | 32 | 5 | 1 |
|  | FDR(%) |  | 1.776 | 0.322 | 0.206 | 0.103 |
| Horsham rain-fed | Average internal infection | 103 | 51 | 29 | 8 | 2 |
|  | FDR(%) |  | 2.020 | 0.355 | 0.129 | 0.052 |
| Horsham irrigated 2017 | Average internal infection | 103 | 61 | 33 | 4 | 1 |
|  | FDR(%) |  | 1.689 | 0.312 | 0.258 | 0.103 |

Table S6. Significant lead SNPs (at ${P<1 x 10}^{-5}$) detected in WGS in a single-trait analysis in integer genotype format are validated using single-trait analysis based on integer format WGS

|  | **Single-trait WGS 012 Discovery *vs* Single-trait WGS 012 validation** | | | | | |
| --- | --- | --- | --- | --- | --- | --- |
| Location in Validation | **Traits** | Number of SNPs in the Discovery set  at P < 1 x 10^-5^ | No. of significant SNPs in the validation set (${at-log}_{10}(p)$ | | | |
|  |  |  | p<10^-2^ | p<10^-3^ | p<10^-4^ | p<10^-5^ |
| Mininera 2016 | Average internal infection | 97 | 37 | 19 | 6 | 1 |
|  | FDR(%) |  | 0.262 | 0.051 | 0.016 | 0.010 |
|  | Emergence count | 104 | 48 | 20 | 4 | 1 |
|  | FDR(%) |  | 0.217 | 0.052 | 0.026 | 0.010 |
| Horsham irrigated 2016 | Average internal infection | 97 | 39 | 17 | 4 | 1 |
|  | FDR(%) |  | 0.249 | 0.057 | 0.024 | 0.010 |
|  | Emergence count | 104 | 49 | 26 | 3 | NA |
|  | FDR(%) |  | 0.212 | 0.040 | 0.035 | NA |
| Horsham rain-fed 2017 | Average internal infection | 32 | 14 | 11 | 3 | 1 |
|  | FDR(%) |  | 0.229 | 0.029 | 0.011 | 0.003 |
|  | Emergence count | 12 | 9 | 5 | 2 | 1 |
|  | FDR(%) |  | 0.133 | 0.024 | 0.006 | 0.001 |
| Horsham irrigated 2017 | Average internal infection | 32 | 13 | 11 | 4 | 1 |
|  | FDR(%) |  | 0.246 | 0.029 | 0.008 | 0.003 |
|  | Emergence count | 12 | 5 | 4 | 2 | NA |
|  | FDR(%) |  | 0.240 | 0.030 | 0.006 | NA |

Table S7. Significant lead SNPs (at ${P<1 x 10}^{-5}$) detected in GBSt – dosage in a single-trait analysis are validated using single-trait GBSt – dosage format

|  | **Single-trait GBS Discovery *vs* Single-trait GBS validation** | | | | | |
| --- | --- | --- | --- | --- | --- | --- |
| Location in Validation | **Traits** | Number of SNPs in the Discovery set  at P < 1 x 10^-5^ | No. of significant SNPs in the validation set (${at-log}_{10}(p)$ | | | |
|  |  |  | p<10^-2^ | p<10^-3^ | p<10^-4^ | p<10^-5^ |
| Mininera 2016 | Average internal infection | 18 | 7 | 3 | 3 | 1 |
|  | FDR(%) |  | 0.257 | 0.060 | 0.006 | 0.002 |
|  | Emergence count | 11 | 6 | 3 | 1 |  |
|  | FDR(%) |  | 0.183 | 0.037 | 0.011 | NA |
| Horsham irrigated 2016 | Average internal infection | 18 | 5 | 2 | 2 | 1 |
|  | FDR(%) |  | 0.360 | 0.090 | 0.009 | 0.002 |
|  | Emergence count | 11 | 4 | 2 | 1 |  |
|  | FDR(%) |  | 0.275 | 0.055 | 0.011 | NA |
| Horsham rain-fed 2017 | Average internal infection | 8 | 4 | 2 | 1 | 1 |
|  | FDR(%) |  | 0.200 | 0.040 | 0.008 | 0.001 |
|  | Emergence count | 4 | 2 | 2 | 1 |  |
|  | FDR(%) |  | 0.200 | 0.020 | 0.004 | NA |
| Horsham irrigated 2017 | Average internal infection | 8 | 3 | 1 | 2 | 1 |
|  | FDR(%) |  | 0.267 | 0.080 | 0.004 | 0.001 |
|  | Emergence count | 4 | 2 | 1 | 1 |  |
|  | FDR(%) |  | 0.200 | 0.040 | 0.004 | NA |

Table S8. List of known resistance genes, QTLs and new genomic regions associated with blackleg disease in canola

| **Chromosome** | **Region** | **Start** | **End** | **No of significant SNPs** | **Gene/QTL** | **Reference or new region** |
| --- | --- | --- | --- | --- | --- | --- |
| ChrA01 | 1 | 703972 | 706447 | 4 |  | *New region* |
| ChrA01 | 2 | 5634661 | 5669730 | 10 |  | *New region* |
| ChrA01 | 3 | 6091049 | 6091095 | 2 | Rlm12 and QTL | [29](#_ENREF_29); [25](#_ENREF_25); [24](#_ENREF_24)^,^[77](#_ENREF_77) |
| ChrA01 | 4 | 19348231 | 19414300 | 14 |  | *New region* |
| ChrA02 | 5 | 3673553 | 3696537 | 92 |  | *New region* |
| ChrA02 | 6 | 4526045 | 4526045 | 1 | LepR1 and QTL | [21](#_ENREF_21), [25](#_ENREF_25); [24](#_ENREF_24) |
| ChrA02 | 7 | 6301998 | 6402898 | 30 |  | [21](#_ENREF_21) |
| ChrA02 | 8 | 6529771 | 6529771 | 1 |  | [21](#_ENREF_21) |
| ChrA02 | 9 | 7462199 | 7462199 | 1 |  | [21](#_ENREF_21) |
| ChrA03 | 10 | 143338 | 159452 | 32 |  | *New region* |
| ChrA03 | 11 | 416959 | 417019 | 2 |  | *New region* |
| ChrA03 | 12 | 1941655 | 1943173 | 8 |  | [25](#_ENREF_25); [24](#_ENREF_24) |
| ChrA03 | 13 | 4802349 | 4803494 | 8 |  | *New region* |
| ChrA03 | 14 | 13641758 | 13641758 | 1 |  | *New region* |
| ChrA03 | 15 | 14142883 | 14145323 | 2 |  | *New region* |
| ChrA03 | 16 | 23416409 | 23416409 | 1 |  | *New region* |
| ChrA03 | 17 | 26273834 | 26273834 | 1 |  | *New region* |
| ChrA04 | 18 | 5266192 | 5266293 | 2 |  | [25](#_ENREF_25); [24](#_ENREF_24) |
| ChrA04 | 19 | 5646553 | 5646553 | 1 |  | *New region* |
| ChrA04 | 20 | 6861138 | 6945247 | 3 |  | *New region* |
| ChrA04 | 21 | 10220228 | 10224546 | 15 |  | *New region* |
| ChrA04 | 22 | 12153549 | 12153549 | 1 |  | *New region* |
| ChrA04 | 23 | 15531921 | 15531921 | 1 |  | *New region* |
| ChrA04 | 24 | 17816506 | 17940112 | 7 |  | *New region* |
| ChrA05 | 25 | 269841 | 273170 | 3 | QTL | [25](#_ENREF_25); |
| ChrA05 | 26 | 2917481 | 2918378 | 7 |  | *New region* |
| ChrA05 | 27 | 4816828 | 4832648 | 3 |  | *New region* |
| ChrA05 | 28 | 20240632 | 20241449 | 2 |  | *New region* |
| ChrA05 | 29 | 22687299 | 22687483 | 2 |  | *New region* |
| ChrA06 | 30 | 5407019 | 5407110 | 2 | LepR4 and QTL | [75](#_ENREF_75) |
| ChrA06 | 31 | 15849189 | 15849189 | 1 |  | [75](#_ENREF_75); [25](#_ENREF_25); [24](#_ENREF_24) |
| ChrA06 | 32 | 19854215 | 19854215 | 1 |  | *New region* |
| ChrA06 | 33 | 23324987 | 23324987 | 1 |  | *New region* |
| ChrA07 | 34 | 1832272 | 1833494 | 20 | Rlm1 and QTL | [14](#_ENREF_14), [25](#_ENREF_25); [24](#_ENREF_24)^,^[77](#_ENREF_77) |
| ChrA07 | 35 | 4924604 | 4924604 | 1 | Rlm3 | [14](#_ENREF_14) |
| ChrA07 | 36 | 10606314 | 10607087 | 7 | Rlm4 | [11](#_ENREF_11) |
| ChrA07 | 37 | 13001036 | 13001036 | 1 | Rlm7 | [13](#_ENREF_13) |
| ChrA07 | 38 | 15755953 | 15755953 | 1 | Rlm9* | - |
| ChrA07 | 39 | 18544724 | 18544724 | 1 | LmFr1 | [12](#_ENREF_12) |
| ChrA07 | 40 | 23522277 | 23523636 | 2 | LMR1 and LEM1 | [12](#_ENREF_12) |

*Note that these resistance genes are known to be ineffective in Australia and therefore we expect that our GWAS signal is for other quantitative resistance in the same region.

Table S8. List of known resistance genes, QTLs and new genomic regions associated with blackleg disease in canola. (cont’d)

| **Chromosome** | **Region** | **Start** | **End** | **No of significant SNPs** | **Gene/QTL** | **Reference or new region** |
| --- | --- | --- | --- | --- | --- | --- |
| ChrA08 | 41 | 11610634 | 11692739 | 28 | Rlm5 and QTL | [76](#_ENREF_76); [25](#_ENREF_25); [24](#_ENREF_24),[77](#_ENREF_77) |
| ChrA08 | 42 | 16215294 | 16215294 | 1 |  | *New region* |
| ChrA08 | 43 | 17787338 | 17796402 | 3 |  | *New region* |
| ChrA08 | 44 | 18108921 | 18110931 | 5 |  | *New region* |
| ChrA08 | 45 | 18816084 | 18816085 | 2 |  | *New region* |
| ChrA09 | 46 | 741861 | 741861 | 1 |  | *New region* |
| ChrA09 | 47 | 8946623 | 9005735 | 3 |  | *New region* |
| ChrA09 | 48 | 12788492 | 12788492 | 1 |  | *New region* |
| ChrA09 | 49 | 25401583 | 25401583 | 1 | QTL | [25](#_ENREF_25); [24](#_ENREF_24)^,^[77](#_ENREF_77) |
| ChrA10 | 50 | 10459187 | 10459493 | 2 | Rlm2*, LepR3* | [22](#_ENREF_22); [16](#_ENREF_16) |
| ChrA10 | 51 | 15386273 | 15386274 | 2 | BLMR1, BLMR2, LepR2* | [92](#_ENREF_92); [93](#_ENREF_93) |
| ChrC01 | 52 | 483267 | 483267 | 1 |  | *New region* |
| ChrC01 | 53 | 8987164 | 8987164 | 1 | QTL | [25](#_ENREF_25); [24](#_ENREF_24)^,^[77](#_ENREF_77) |
| ChrC03 | 54 | 20168080 | 20168080 | 1 | - | *New region* |
| ChrC03 | 55 | 49259071 | 49265050 | 10 |  | *New region* |
| ChrC04 | 56 | 795412 | 797105 | 10 |  | *New region* |
| ChrC04 | 57 | 3846333 | 3846397 | 3 |  | *New region* |
| ChrC04 | 58 | 4791635 | 4794297 | 10 |  | *New region* |
| ChrC04 | 59 | 6041535 | 6041535 | 1 |  | *New region* |
| ChrC04 | 60 | 6459981 | 6460584 | 3 |  | *New region* |
| ChrC04 | 61 | 26090780 | 26090780 | 1 |  | *New region* |
| ChrC04 | 62 | 47331241 | 47331241 | 1 | QTL | [25](#_ENREF_25) |
| ChrC05 | 63 | 7807600 | 7807620 | 2 |  | *New region* |
| ChrC06 | 64 | 15935066 | 15936299 | 16 |  | *New region* |
| ChrC06 | 65 | 25300828 | 25442922 | 3 |  | *New region* |
| ChrC06 | 66 | 28340821 | 28341208 | 10 |  | *New region* |
| ChrC06 | 67 | 28743171 | 28743171 | 1 |  | *New region* |
| ChrC06 | 68 | 32555569 | 32721780 | 80 |  | *New region* |
| ChrC06 | 69 | 32861289 | 33037050 | 4 |  | *New region* |
| ChrC06 | 70 | 33943262 | 33943262 | 1 |  | *New region* |
| ChrC06 | 71 | 34304258 | 34389934 | 40 |  | *New region* |
| ChrC06 | 72 | 34982347 | 35030651 | 2 | QTL | [25](#_ENREF_25); [24](#_ENREF_24)^,^[77](#_ENREF_77) |
| ChrC07 | 73 | 25730360 | 25730439 | 3 | Rlm6 and QTL | [76](#_ENREF_76); [25](#_ENREF_25); [24](#_ENREF_24)^,^[77](#_ENREF_77) |
| ChrC07 | 74 | 33086130 | 33086132 | 2 |  | *New region* |
| ChrC07 | 75 | 42187529 | 42187534 | 2 |  | *New region* |
| ChrC07 | 76 | 42436493 | 42438935 | 5 |  | *New region* |
| ChrC08 | 77 | 30825427 | 30838470 | 51 |  | *New region* |
| ChrC08 | 78 | 33361777 | 33361873 | 3 | QTL | [25](#_ENREF_25); [24](#_ENREF_24)^,^[77](#_ENREF_77) |
| ChrC09 | 79 | 41119643 | 41194171 | 63 |  | *New region* |

*Note that these resistance genes are ineffective in Australia and therefore we expect that our GWAS signal is for other quantitative resistance in the same region.

Table S9. List of SNP effect annotation (Effect Classes count) across 19 *Brassica napus* L. chromosome

|  | **SNP effect annotation (Effect Classes/chromosome)** | | | | | | |
| --- | --- | --- | --- | --- | --- | --- | --- |
| **Chr.** | **Intragenic** | **Intergenic** | **Intron** | **Synonymous coding** | **Non-Synonymous coding** | **Stop gained** | **Stop lost** |
| ChrA01 | 9 | 11 | 7 | 2 | 1 |  |  |
| ChrA02 | 9 | 62 | 22 | 21 | 10 | 1 | - |
| ChrA03 | 6 | 24 | 10 | 3 | 9 | 1 | 2 |
| ChrA04 | 3 | 17 | 7 | - | 3 | - | - |
| ChrA05 | - | 5 | 9 | - | 3 | - | - |
| ChrA06 | - | - | 1 | 1 | 4 | - | - |
| ChrA07 | 1 | 23 | 5 | 3 | 1 | - | - |
| ChrA08 | 8 | 8 | 11 | 3 | 9 | - | - |
| ChrA09 | - | 2 | 3 | - | 1 | - | - |
| ChrA10 | - | 2 | - | - | 2 | - | - |
| ChrC01 | - | - | 1 | - | 1 | - | - |
| ChrC03 | 1 | 2 | 8 | - | - | - | - |
| ChrC04 | 5 | 7 | 10 | 5 | 2 | - | - |
| ChrC05 |  | - | - | - | 2 | - | - |
| ChrC06 | 17 | 84 | 41 | 9 | 6 | - | - |
| ChrC07 | - | 6 | 2 | 1 | 2 | - | - |
| ChrC08 | - | 46 | 7 | 1 | - | - |  |
| ChrC09 | 7 | 32 | 14 | 4 | 6 | - | - |

Intron = between exons; intergenic = between genes; stop-gained or stop-lost = (within an exon and translated, non-stop codon changed to stop codon or stop codon changed to non-stop codon)
